# An encodable amino acid for targeted photocatalysis†

**DOI:** 10.1039/d4sc08594a

**Published:** 2025-01-28

**Authors:** Man Sing Wong, Utsa Karmakar, Marco Bertolini, Abigail E. Reese, Lorena Mendive-Tapia, Marc Vendrell

**Affiliations:** a Centre for Inflammation Research, The University of Edinburgh EH16 4UU Edinburgh UK; b IRR Chemistry Hub, Institute for Regeneration and Repair, The University of Edinburgh EH16 4UU Edinburgh UK marc.vendrell@ed.ac.uk

## Abstract

Photocatalysts are excellent scaffolds for the light-mediated control of bioactive molecules. Current photocatalytic structures are not compatible with genetic encoding and therefore cannot be directly incorporated into the sequences of native proteins. Herein, we developed new amino acids incorporating Si-rosamine photocatalytic units, and introduced them *via* aminoacylation of tRNAs into specific positions of different proteins to enable targeted photocatalytic reactions in defined populations of immune cells.

## Abstract 2

New Si-rosamine amino acids were site-specifically introduced into whole protein structures. The resulting conjugates were employed for targeted photocatalytic reactions in subpopulations of immune cells.

## Graphical abstract

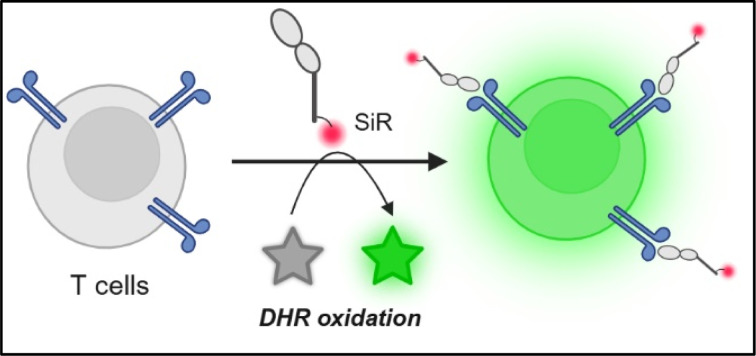

## Introduction

Photocatalytic reactions have multiple uses in chemical biology, life sciences and the pharmaceutical industry. Recent examples include their application to proximity labelling of receptors and proteins for studying protein–protein interactions and signalling pathways.^1–4^ Numerous photocatalysts have been described in the literature, and their molecular structures range from transition metal complexes^5^ and nanoparticles^6^ to organic chromophores.^7^ Upon light irradiation, organic photocatalysts generate reactive oxygen species (ROS) which can accelerate the decaging of drugs and/or the release of bioactive molecules.^8^ However, one major challenge in the application of organic photocatalysts to biological systems is the design of chemical strategies that enable their targeted delivery to specific subpopulations of cells.

**Fig. 1:**
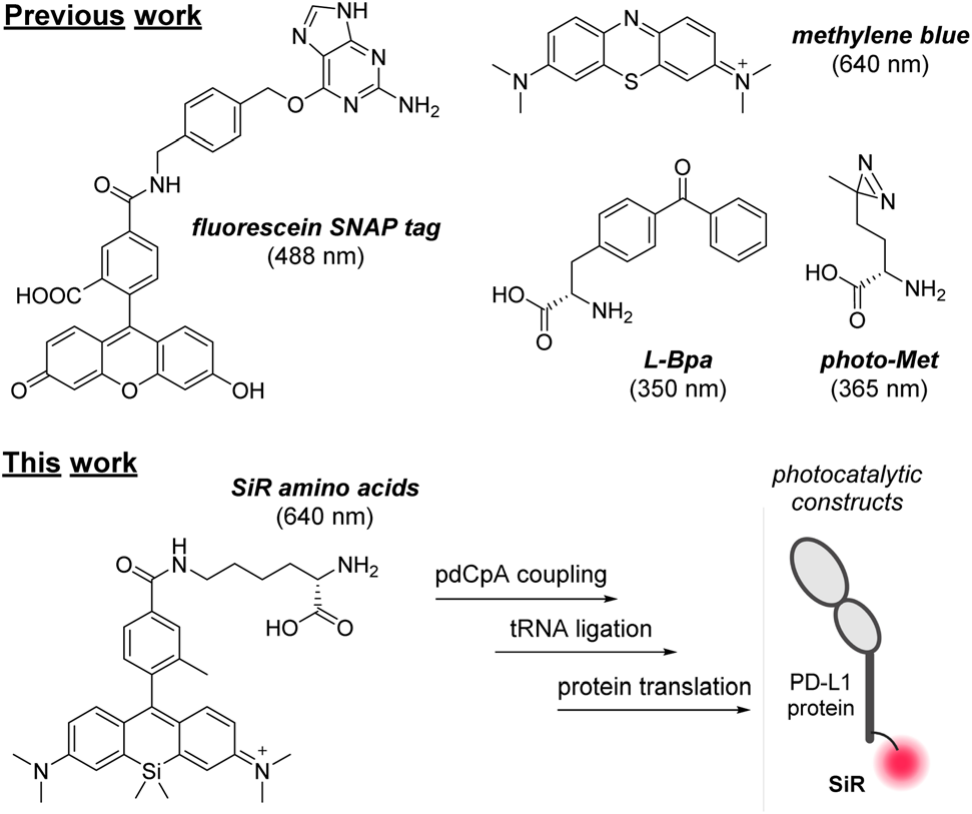
A diverse range of photocatalysts and photoreactive amino acids. Top panel: chemical structures and excitation wavelengths of reported probes, including fluorescein-SNAP tag, methylene blue and the encodable amino acids L-Bpa and photo-Met. Bottom panel: new SiR amino acids for incorporation into proteins *via* cell-free translation.

Several UV-active small molecules including benzophenones and diazirines have been used in photoaffinity and light-mediated labelling studies.^9–12^ Some of these probes require activation at short excitation wavelengths (*e.g.*, 350 nm for benzophenones, 365 nm for diazirines, Fig. 1), which can cause cellular damage. Metal-based photocatalysts (*e.g.*, ruthenium and iridium) and nanoparticles can be conjugated to proteins for targeted delivery;^6,13,14^ however, these are large structures that can impede the molecular recognition of target receptors. We envisaged that the adaptation of smaller organic photocatalysts activated at red/near-infrared (NIR) excitation wavelengths into protein structures could generate targeted photocatalysts with minimal phototoxicity.

The Fox group and others have reported red-activated photocatalysts, including unmodified methylene blue and more recently silicon-rhodamine (SiR) probes for light-induced oxidation of dihydrotetrazines to bioorthogonal tetrazines for targeted imaging and drug delivery.^15–17^ Other photocatalysts have been derivatised as ligands for self-labelling proteins (*e.g.*, fluorescein SNAP tag, Fig. 1) for targeted drug release.^18^ Our group has recently optimised methods to incorporate fluorescent amino acids at selected positions of different proteins,^19–22^ thus we decided to explore the development of photocatalytic amino acids that could be encoded into protein structures for the first time.

SiR are photostable far-red chromophores with broad applications in chemical biology.^23,24^ In this work, we have synthesised a collection of SiR photocatalyst amino acids for protein derivatisation (Fig. 1). We ligated selected SiR amino acids to tRNA molecules for cell-free translation and site-selective incorporation into different proteins, including the first example of a programmed death ligand 1 (PD-L1) analogue for targeted photocatalysis in T cells expressing PD-1 receptors. This new chemical tool will accelerate the future design of artificial proteins for targeted photocatalysis.

## Results and discussion

### Synthesis and *in vitro* evaluation of a collection of new SiR photocatalytic amino acids

Given the suitable properties of the SiR core for building far-red photocatalysts,^25,26^ we synthesised a small collection of SiR-based amino acids and assessed their potential to be directly encoded into proteins. We envisaged that the side chains could affect their compatibility with genetic code expansion, and considered 5 amino acids (*i.e.*, lysine (Lys), 4-aminophenylalanine (aPhe), 2,3-diaminopropionic acid (Dap), 2,4-diaminobutyric acid (Dab), and ornithine (Orn)). The systematic evaluation of this collection allowed us to interrogate how variations in alkyl/aryl groups and chain length influenced their optical properties and compatibility with tRNA aminoacylation and protein translation.

**Fig. 2:**
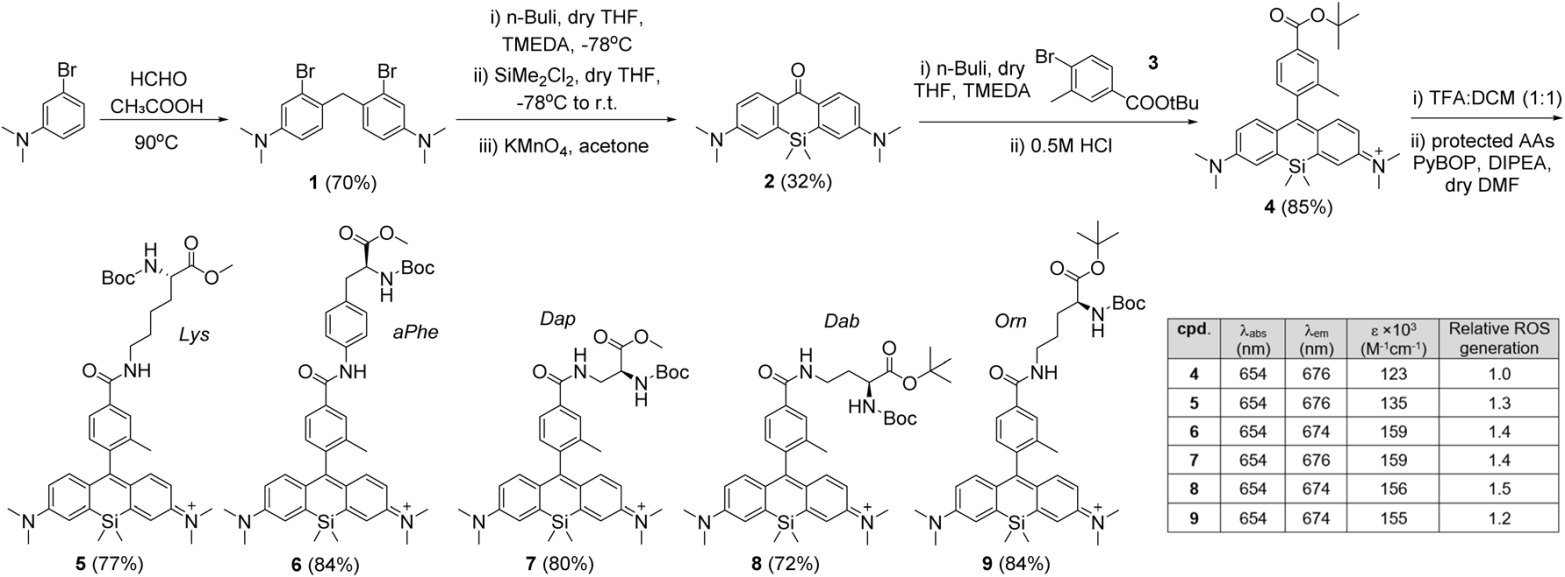
Synthetic scheme for the preparation of SiR photocatalytic amino acids. Optical characterisation for compounds 4–9 was performed in EtOH (10 μM compounds). Extinction coefficients were measured at 650 nm. ROS generation for the amino acids (5–9) was determined by monitoring the decrease in absorbance of DPBF (410 nm) after illumination at 640 nm (0.4 mW cm^−2^, 4 min) and referred to that of the non-amino acid SiR compound 4 (*n* = 3).

We decided to conjugate the previously reported SiR-2-Me scaffold^27^ to the different amino acids through an amide bond at position 5 (*i.e.*, to avoid any potential steric hindrance). The synthesis of SiR-based amino acids is shown in Fig. 2. Briefly, we first prepared 4,4′-methylenebis(3-bromo-*N*,*N*-dimethylaniline) (1) from the commercial precursor 3-bromo-*N*,*N*-dimethylaniline, followed by a lithium–halogen exchange reaction to afford the Si-containing tetramethylxanthone 2. Next, we performed lithiation of *tert*-butyl 4-bromo-3-methyl benzoate (3) and reacted it with compound 2 to form the SiR scaffold as a protected ester (4).^28^ Compound 4 was treated with trifluoroacetic acid (TFA) to render the corresponding carboxylic acid, which was coupled to the protected amino acids using benzotriazol-1-yloxytripyrrolidinophosphonium hexafluoro-phosphate (PyBOP) in basic media in DMF. All amino acids (compounds 5–9, Fig. 2) were isolated by preparative HPLC chromatography in good yields and purities (see Supplementary Information† for full synthetic and characterisation details).

Upon completing the chemical synthesis, we evaluated the optical properties of the amino acids 5–9 (Fig. 2 and Table S1†) and compared them to the SiR precursor 4. As expected, all SiR derivatives demonstrated similar excitation wavelengths ∼650 nm in the far-red range of the visible spectrum, with extinction coefficients around 10^5^ M^−1^ cm^−1^ (Fig. 2 and S1†). Furthermore, we examined their capacity to generate ROS -including singlet oxygen upon far-red light irradiation at 640 nm. For this experiment, we monitored the oxidation of 1,3-diphenylisobenzofuran (DPBF) after illumination in the presence of all SiR compounds 4–9 and the commercial methylene blue as a far-red photocatalyitic positive control. As shown in Fig. 2 and S2,† compounds 5–9 showed similar ROS generation to the precursor compound 4, confirming that the integration of the SiR scaffold into different amino acid structures did not affect their potential performance as photocatalysts.

### Site-specific incorporation of photocatalytic amino acids into proteins

**Fig. 3:**
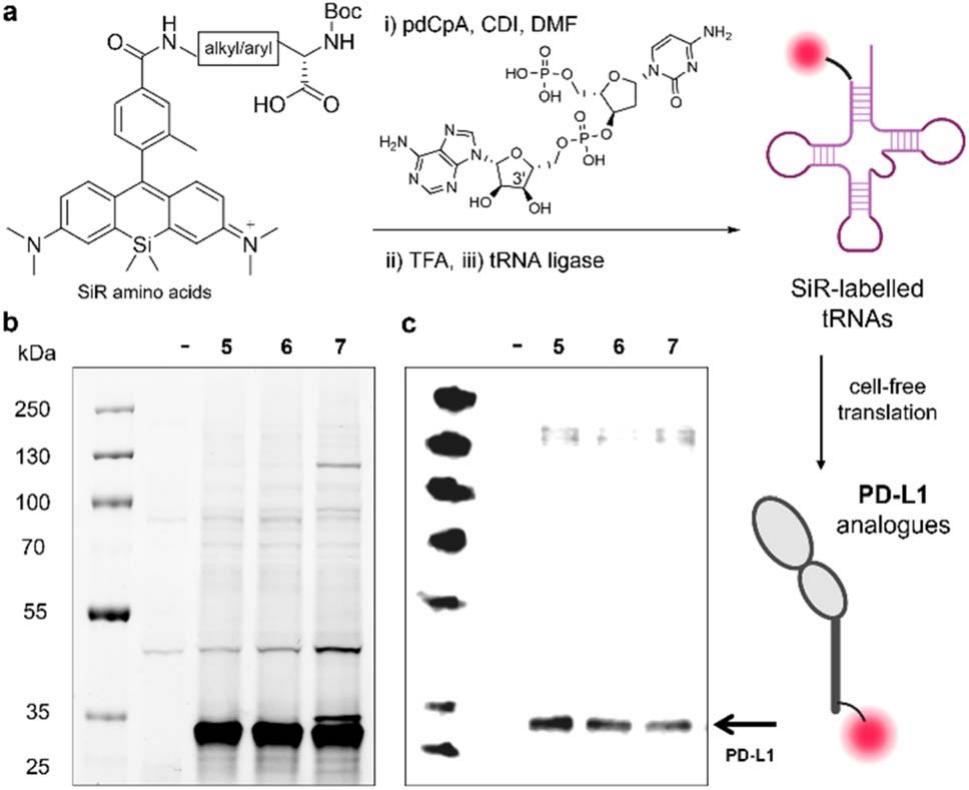
Site-specific incorporation of SiR amino acids into PD-L1. (a) Schematic illustration of pdCpA coupling to SiR amino acids, followed by Boc removal, tRNA ligation and cell-free translation. (b) Representative SDS-PAGE in-gel fluorescence analysis of SiR-labelled PD-L1 proteins after incorporation of the amino acids 5, 6 and 7 (654/676 nm) compared to a negative reaction control with no plasmid DNA (–). (c) Representative western blot analysis of SiR-labelled proteins using an anti-PD-L1 antibody. Gel images were analysed using Image Lab.

Cell-free strategies for protein expression have been reported as alternatives to *in vivo* systems that require orthogonal aaRS/tRNA synthetases for non-canonical amino acids (ncAAs).^29^ These systems enable introduction of relatively large ncAAs into the sequences of proteins, first by aminoacylation of tRNA molecules and followed by ribosome-mediated incorporation using suitable translation systems. Among the different strategies for tRNA aminoacylation, we employed the chemical aminoacylation approach^30^ whereby our SiR amino acids were coupled to the 3′-hydroxyl group of 5′-phospho-2′-deoxyribocytidylylriboadenosine dinucleotide (pdCpA) followed by enzymatic ligation to truncated tRNA (Fig. 3). We envisaged that SiR-tRNAs could be then introduced into proteins by cell-free translation.

In order to prepare pdCpA derivatives from compounds 5–9, we hydrolysed the amino acids under basic conditions and conjugated them to pdCpA with 1,1′-carbonyldiimidazole (CDI) as the coupling agent.^31^ The extent of conjugation varied slightly between amino acids, with compounds 5–7 rendering pdCpA analogues with the highest purities >90% (Fig. S3–S5 and Table S2†). Therefore, we used these conjugates further, treated them with TFA to remove the Boc protecting groups, and ligated the resulting SiR-pdCpA to tRNA *via* enzymatic ligation with T4 RNA ligase 1 (Fig. 3a).

Having successfully ligated the SiR amino acids 5–7 to tRNA, we next investigated their ability to be incorporated into proteins. For these experiments, we selected PD-L1 as a model protein. PD-L1 is a transmembrane protein overexpressed in cancer cells,^32–34^ which acts as a ligand of the co-inhibitory programmed death receptor 1 (PD-1)^35^ and plays an important role in maintaining T cell tolerance and autoimmunity.^36^ We attempted the cell-free translation of PD-L1 using the 3 tRNA conjugates containing the amino acids 5, 6 and 7. In these experiments, we used the NEB Express cell-free *E. coli* protein synthesis system supplemented with the SiR-tRNAs, a NEB plasmid containing the coding sequence for the bioactive fragment of PD-L1 (*i.e.*, Phe19-Thr239, 27 kDa) with an amber stop (UAG) codon at position 2 and a release factor 1 inhibitor (Api137) to ensure full reassignment of UAG. Next, we assessed the production of labelled PD-L1 conjugates by in-gel fluorescence analysis and western blot using an anti-PD-L1 antibody (Fig. 3b and c). Our results showed bright fluorescent bands at the molecular weight of PD-L1, indicating successful incorporation of the SiR amino acids 5–7. Notably, in all cases we observed matching bands in the western blot analysis indicating that the main constructs were recognised by an anti-PD-L1 antibody (Fig. 3bc). Altogether, this approach represents the first example of site-specific and ribosome-mediated incorporation of SiR-based amino acids into large protein structures.

### The amino acid 5 is an effective photocatalyst and it can be introduced into different proteins

Lys-based unnatural amino acids have been genetically encoded into multiple proteins in prokaryotic and mammalian cells.^37,38^ Given the efficient incorporation of the Lys amino acid 5 into proteins, we further studied its application in photocatalysis. The synthesis of the amino acid 5 is scalable to amounts in the 50-100 mg scale, thus providing sufficient material for subsequent spectroscopic and biological studies.

**Fig. 4:**
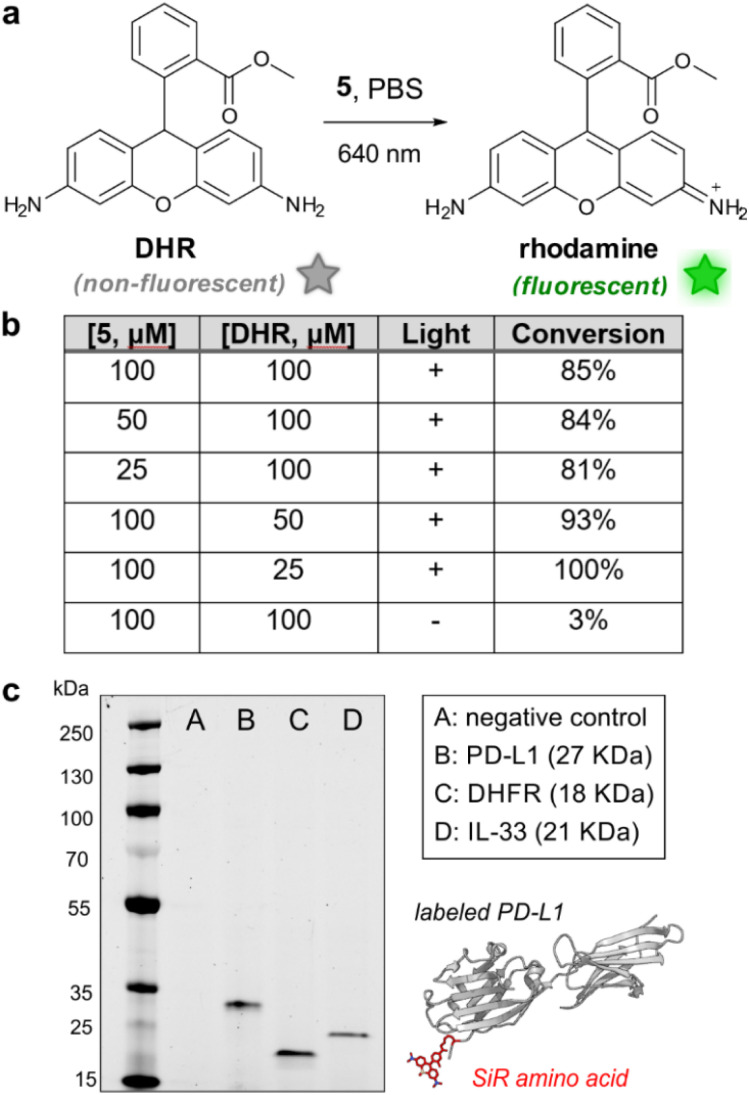
Amino acid 5 is an effective photocatalyst for the oxidation of the profluorophore DHR. (a) Photocatalytic oxidation of the non-fluorescent DHR to the rhodamine 123 fluorophore. (b) Summary of the reaction conditions and HPLC conversions of the photocatalytic oxidation of DHR using the amino acid 5 (extended results in Fig. S6†). (c) Representative SDS-PAGE in-gel fluorescence analysis of the PD-L1, DHFR and IL-33 proteins after incorporation of the amino acid 5 (654/676 nm), including a negative reaction control with no plasmid DNA (–). (Inset) Schematic cartoon of the PD-L1 construct with incorporation of the amino acid 5 at the N-terminus (PDB: 3BIK). DHR: dihydrorhodamine.

First, we evaluated the photocatalytic activity of 5 using the profluorophore dihydrorhodamine 123 (DHR, Fig. 4a). DHR is a non-fluorescent compound that can be photocatalytically oxidised by ROS to afford the fluorophore rhodamine 123.^39^ We analysed the oxidation of DHR in PBS buffer in the presence of compound 5 and light irradiation (640 nm, 7–10 mW cm^−2^) and observed conversion rates over 80% by HPLC analysis (Fig. 4b). Notable fluorescence fold increases were observed due to the generation of rhodamine 123 and we confirmed that the photocatalytic oxidation of DHR did not take place in the absence of far-red illumination (Fig. S6†). Similar fluorescence fold increases were observed with the commercial methylene blue, featuring the utility of amino acid 5 as a far-red photocatalyst (Fig. S6†). Furthermore, we also employed the amino acid 5 to effectively uncage a doxorubicin analogue including a ROS-cleavable boronate protecting group^40^ (Fig. S7†). Altogether, these results assert the ability of the Lys-based SiR amino acid 5 to photocatalytically uncage both profluorophores and prodrugs, and its potential application in different photocatalytic reactions.

Finally, we evaluated the versatility of the amino acid 5 for genetic code expansion and examined the cell-free translation of different small proteins, namely dihydrofolate reductase (DHFR, 18 kDa) and interleukin 33 (IL-33, 21 kDa). These experiments were performed under similar experimental conditions to those utilised for PD-L1 expression. As shown in Fig. 4c, we observed bright fluorescent bands at the right molecular weights for all three proteins, which confirms the modularity of Lys-based SiR amino acids as encodable building blocks for protein diversification.

### A photocatalytic analogue of PD-L1 protein targets subpopulations of PD-1 expressing T cells

The PD-L1/PD-1 axis serves as an immune checkpoint to inhibit anti-tumour T cell immunity by hindering T cell effector functions such as cell proliferation, cytokine production and cytotoxicity. PD-1 is highly expressed in activated T cells, and in haematological malignancies PD-1 is overexpressed in T cells driving them to the exhaustion stage.^41^ Therefore, the PD-L1/PD-1 axis represents an attractive target to modulate cancer cell-T cell interactions.

In order to scale up the synthesis of a photocatalytic PD-L1 analogue, we prepared a recombinant derivative of PD-L1 where an unnatural azidoPhe residue was incorporated at position 2 and conjugated to a bicyclo[6.1.0]non-4-yne (BCN)-containing derivative of the amino acid 5 (compound 11, see ESI† for synthetic details and characterisation data). This approach rendered milligrams of the PD-L1-11 protein in good purity for spectroscopy cell-based assays (Fig. S8†). Similar to the amino acid 5, PD-L1-11 was able to photocatalytically oxidise the profluorophore DHR under far-red illumination (640 nm, 10 mW cm^−2^) as observed by notable increases in fluorescence emission (Fig. S9†).

Next, we proceeded to perform cell-based assays to examine the application of the PD-L1-11 protein for targeted photocatalysis in PD-1 expressing T cells. T cells were isolated from peripheral blood of healthy volunteers and were stimulated with CD3-and CD28-coated beads as previously reported.^42,43^ First, we employed flow cytometry to confirm significantly different expression of PD-1 receptors in non-stimulated T cells (PD-1 negative) *vs.* stimulated T cells (PD-1 positive) by anti-PD-1 antibody staining (Fig. S10 and S11†). Second, we confirmed that the PD-L1-11 protein retained the ability to recognise PD-1 receptors and preferentially bound to PD1-expressing stimulated T cells (Fig. S12†). These results confirm that the incorporation of Lys-based SiR photocatalysts at the position 2 of PD-L1 did not affect their photocatalytic potential of the SiR core or the ability of the protein ligand to bind its cognate receptor.

**Fig. 5:**
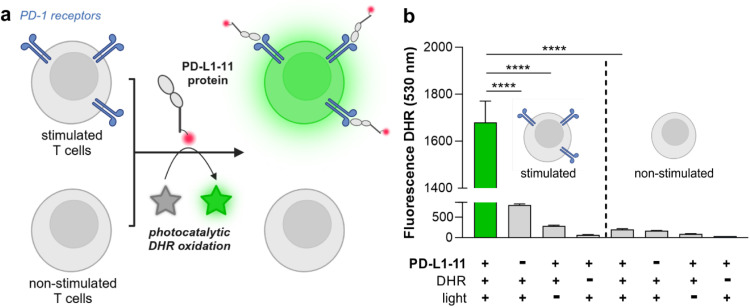
A photocatalytic PD-L1 analogue targets subpopulations of PD-1 expressing stimulated T cells. (a) Schematic cartoon of the photocatalytic oxidation of DHR (500 nM) in PD-1 expressing stimulated T cells (but not in PD-1-negative non-stimulated T cells) mediated by the PDL1-11 protein (5 μM) and far-red light illumination (640 nm, 10 mW cm^−2^). (b) Flow cytometry analysis of DHR oxidation -by measuring the fluorescence emission of rhodamine 123 at 530 nm-in live stimulated T cells and live non-stimulated T cells. Valued presented as means ± SD (*n* = 3). *P* values were determined by ne-way ANOVA (**** for *p* < 0.0001). DHR: dihydrorhodamine.

Finally, we evaluated the photocatalytic activity of PD-L1-11 in subpopulations of live T cells that were non-stimulated (low PD-1 receptor expression) or stimulated (high PD-1 receptor expression) (Fig. 5a). For these experiments, both cell populations were incubated first with PD-L1-11 (5 μM), then with the profluorophore DHR (500 nM) followed by far-red light illumination (640 nm, 10 mW cm^−2^). We employed flow cytometry to analyse the extent of photocatalytic DHR oxidation by measuring the fluorescence emission of uncaged rhodamine at 530 nm and observed that stimulated PD-1-expressing T cells showed significantly brighter fluorescence emission than non-stimulated T cells (Fig. 5b). We also observed a significant difference in fluorescence emission in stimulated T cells that had been incubated with PD-L1-11 and DHR but were not illuminated with red light (Fig. 5b). Viability assays in T cells confirmed that DHR or the illumination regime had no toxic effects (Fig. S13 and S14†).

## Conclusions

In summary, we have developed new SiR-based photocatalyst amino acids and optimised their site-specific incorporation into proteins. We synthesised a small collection of SiR amino acids with variable side chains and confirmed their ability to generate ROS under illumination with far-red light (640 nm). The amino acids were successfully ligated to tRNA -via chemical amino acylation using pdCpA-and then incorporated into 3 different proteins (PD-L1, DHFR and IL-33) using cell-free translation. We further proved that Lys-containing amino acids can photocatalytically oxidize the profluorophore DHR on their own as well as after incorporation into protein structures. Finally, we generated the first example of a PD-L1 protein analogue and demonstrated its photocatalytic activity in specific subpopulations of stimulated T cells expressing PD-1 receptors (but not in non-stimulated T cells lacking PD-1 receptors). This new chemical tool will accelerate the chemical design of new artificial proteins for targeted photocatalysis.

## Data availability

The data supporting this article have been included as part of the ESI.†

## Author contributions

M. S. W.: investigation, methodology and writing original draft; U. K.: investigation, methodology; M. B.: investigation; A. E. R.: investigation; L. M. T.: supervision, review and editing; M. V.: conceptualisation, funding acquisition, supervision, writing, review and editing.

## Conflicts of interest

There are no conflicts to declare.

## Supplementary Material

SC-OLF-D4SC08594A-s001
