# Supplementary material for: An encodable amino acid for targeted photocatalysis
Source: Chem Sci. 2025 Jan 28;16(10):4360–5. doi: 10.1039/d4sc08594a (PMC11793017; doi:10.1039/d4sc08594a)
Supplement: SC-016-D4SC08594A-s001 [file SC-016-D4SC08594A-s001.pdf]

Supplementary Information (SI) for Chemical Science.
This journal is © The Royal Society of Chemistry 2025

Electronic Supporting Information

An encodable amino acid for targeted photocatalysis

Table of Contents

1. Materials and Methods

2. Experimental Procedures

3. Supplementary Figures and Tables

4. NMR Spectra

5. Supplementary References

1. Materials and Methods

Commercially available reagents were used without further purification. Solvents were purchased as AR grade. Column chromatography was performed using silica gel Merck 60 (particle size 0.040-0.063 mm), with indicated eluent systems. Protected amino acid esters were obtained from AA Blocks (Boc-Dap-OMe HCl and Boc-Phe(4-NH_2)-OMe), Aaron Chemicals LLC (Boc-Dab-OtBu and Boc-Orn-OtBu), Bachem (Boc-Lys-OMe HCl) and Fluorochem (Nα-Ac-Lys-OMe HCl). 3-Bromo-*N*,*N*-dimethylaniline, 4-iodo-3-methylbenzoic acid, trifluoroacetic acid and propargylamine were purchased from Fluorochem. Formaldehyde, 2.5 M n-butyllithium in hexane, tetramethylethylenediamine, dichlorodimethylsilane, potassium permanganate, *N*,*N*-diisopropylethylamine were purchased from Sigma Aldrich. Benzotriazol-1-yloxytripyrrolidinophosphonium hexafluorophosphate was purchased from Alfa Aesar Chemicals. Acetic acid was purchased from Fisher Scientific. Spectroscopic data was measured on a Synergy HT spectrophotometer (Biotek) and analysed using GraphPad Prism 10.0. Preparative HPLC was performed using an Agilent Technologies 1260 system with a Kinetex C18 150 × 21.2 mm column, in H_2O (0.1% CF_3COOH) and ACN (0.1% CF_3COOH) eluents and 20.0 mL min^{-1} flow rate. Synthesis reactions and products were monitored by HPLC-MS using a HPLC Agilent Technologies 1200 with a Kinetex C18 50 × 4.6 mm column and a diode array detector, in H_2O (0.1% formic acid) and ACN (0.1% formic acid) and 1.0 mL min^{-1} flow rate. The MS detector was configured with an electrospray ionisation source and nitrogen as the nebuliser

gas. ^{1}H NMR and ^{13}C NMR spectra were recorded using Bruker Avance III spectrometer operated at 500 MHz and 126 MHz respectively. Chemical shifts were reported in ppm on the δ scale relative to a residual solvent ($CDCl_3$: δ = 7.26 ppm for ^{1}H NMR and 77.2 ppm for ^{13}C NMR). Multiplicities are referred to the following abbreviations: s = singlet, d = doublet, t = triplet, dd = doublet doublets, ddd = double double doublet, dt = double triplet, q = quartet and m = multiplet. HRMS were collected using an electrospray ionisation (ESI) ThermoElectron MAT 900.

2. Experimental Procedures

Chemical synthesis

4,4'-Methylenebis(3-bromo-*N*,*N*-dimethylaniline) (1)

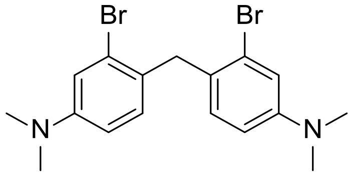

To a mixture of 3-bromo-*N*,*N*-dimethylaniline (6.0 g, 30.0 mmol) in acetic acid (60 mL) was added 37% p-formaldehyde (1.4 mL, 19.5 mmol). The reaction was refluxed for 3 hours. Once the reaction was completed, the solvent was removed under reduced pressure and basified with saturated aqueous $NaHCO_3$ (pH 8). The aqueous layer was extracted with CH_2Cl_2 (70 mL × 3). The organic fractions were combined, washed with brine and dried with $MgSO_4$. The mixture was filtered and concentrated under reduced pressure. The crude material was purified by normal-phase chromatography using CH_2Cl_2:hexane (8:2) to yield the product as a white solid (4.3 g, 70%).

^{1}H NMR (500 MHz, $CDCl_3$): δ 6.94 (d, *J* = 2.7 Hz, 2H), 6.85 (d, *J* = 8.6 Hz, 2H), 6.58 (dd, *J* = 8.6, 2.7 Hz, 2H), 4.00 (s, 2H), 2.91 (s, 12H) ppm.

^{13}C NMR (126 MHz, $CDCl_3$): δ 150.2, 130.9, 127.3, 125.8, 116.4, 112.0, 40.7, 40.0 ppm.

HRMS (ESI) calcd. for $C_{17}H_{21}Br_2N_2$ $[M+H]^+$: 411.0066; found, 411.0061.

3,7-bis(dimethylamino)-5,5-dimethyldibenzo[*b,e*]silin-10(5*H*)-one (2)[1]

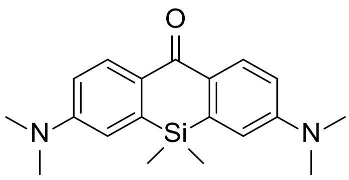

A solution of 4,4'-methylenebis(3-bromo-*N*,*N*-dimethylaniline) (2.0 g, 4.9 mmol) in anhydrous THF (15 mL) was cooled down at -78°C under nitrogen atmosphere. To a mixture of anhydrous THF (20 mL) and TMEDA (2.9 mL, 19.6 mmol) at -78°C under nitrogen atmosphere was added n-butyllithium (2.5 M in hexane, 10 mL) dropwise. After 5 minutes, the n-Buli mixture (25.8 mL, 19.6 mmol) was added dropwise into the dimethylaniline mixture and the mixture was stirred at -78°C for 30 minutes. $SiMe_2Cl_2$ (1.2 mL, 9.8 mmol) in anhydrous THF (6.0 mL) was added into the mixture under nitrogen atmosphere. The mixture was allowed to stir at r.t. overnight and quenched with 2 M HCl (24 mL). The aqueous layer was extracted with CH_2Cl_2 (70 mL×3). The organic fractions were combined, washed with brine and dried with $MgSO_4$. The mixture was filtered and concentrated under reduced pressure. To the mixture in acetone (40 mL) at 0°C was added $KMnO_4$ (2.3 g, 14.6 mmol) over 30 minutes and the mixture was stirred at r.t. for 3 hours. Once the reaction was completed, the solid was filtered, and solvents were removed under reduced pressure. The crude material was purified by flash column chromatography using hexane:EtOAc:CH_2Cl_2 (5:1:1) to isolate the product as a yellow solid (503 mg, 32%).

^{1}H NMR (500 MHz, $CDCl_3$): δ 8.40 (d, *J* = 8.9 Hz, 2H), 6.87 (dd, *J* = 8.9, 2.6 Hz, 2H), 6.85 (d,

J = 2.6 Hz, 2H), 3.10 (s, 12H), 0.47 (s, 6H) ppm.

^{13}C NMR (126 MHz, $CDCl_3$): δ 185.3, 151.4, 140.7, 131.8, 114.85, 113.6, 40.4, -0.9 ppm.

HRMS (ESI) calcd. for $C_{19}H_{24}N_2OSiNa$ $[M+Na]^+$: 347.1550; found, 347.1537.

tert-Butyl 4-iodo-3-methylbenzoate (3)

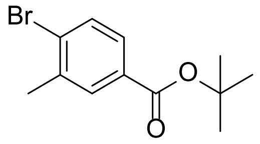

To a mixture of 4-bromo-3-methylbenzoic acid (2.0 g, 9.3 mmol) in *tert*-butyl alcohol (46 mL) was added di-*tert*-butyl decarbonate (6.1 g, 27.9 mmol) and 4-dimethylaminopyridine (0.3 g, 2.8 mmol). The mixture was allowed to stir overnight under nitrogen at 40°C. The solution was diluted with water (150 mL) and extracted with ethyl acetate (100 mL × 3). The organic fractions were combined, washed with brine and dried with $MgSO_4$. The mixture was filtered and concentrated under reduced pressure. The crude material was purified by flash column chromatography on silica gel using hexane:EtOAc (9:1) to isolate the product as an orange liquid (1.98 g, 78%).

^{1}H NMR (500 MHz, $CDCl_3$): δ 7.83 (d, *J* = 2.4 Hz, 1H), 7.64 (dd, *J* = 8.3, 2.4 Hz, 1H), 7.57 (d, *J* = 8.3 Hz, 1H), 2.44 (s, 3H), 1.59 (s, 9H) ppm.

^{13}C NMR (126 MHz, $CDCl_3$): δ165.4, 138.1, 132.4, 131.7, 131.3, 129.9, 128.3, 81.5, 28.3, 23.0 ppm.

HRMS (ESI) calcd. for $C_{12}H_{16}BrO_2$ $[M+H]^+$: 271.0328; found, 271.0338.

***N*-(10-(4-(*tert*-butoxycarbonyl)-2-methylphenyl)-7-(dimethylamino)-5,5-dimethyldibenzo[*b*,*e*]silin-3(5*H*)-ylidene)-*N*-methylmethanaminium chloride (4)**[1]

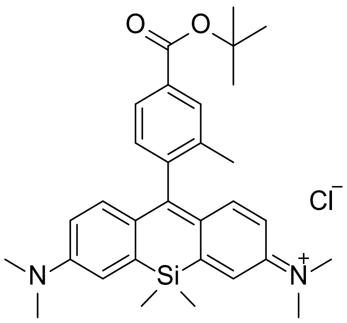

A solution of compound **3** (0.3 g, 1.5 mmol) in anhydrous THF (6 mL) was cooled down to -78°C under nitrogen atmosphere. To a mixture of anhydrous THF (2 mL) and TMEDA (220 μL, 1.5 mmol) at -78°C under nitrogen atmosphere was added n-butyllithium (2.5 M in hexane, 1 mL) dropwise. After 5 minutes, the mixture was added dropwise into the solution of compound **3** and allowed to stir at -78°C for 30 minutes. Then, compound **2** (50 mg, 0.15 mmol) in anhydrous THF (3 mL) was added to the mixture under nitrogen atmosphere and stirred at -78°C for 30 minutes. The reaction mixture was diluted with CH_2Cl_2 (10 mL) and quenched with 0.5 M HCl (10 mL). Organic fractions were extracted with CH_2Cl_2 (20 mL × 3), combined, washed with brine and dried with $MgSO_4$. The crude material was purified by flash column chromatography on silica gel in CH_2Cl_2:MeOH (9:1) to isolate the product as a blue solid (70 mg, 85%).

^{1}H NMR (500 MHz, $CDCl_3$): δ 7.98 (s, 1H), 7.96 (d, *J* = 7.8 Hz, 1H), 7.20 (d, *J* = 2.8 Hz, 2H), 7.17 (d, *J* = 7.8 Hz, 1H), 7.00 (d, *J* = 9.6 Hz, 2H), 6.57 (dd, *J* = 9.6, 2.8 Hz, 2H), 3.35 (s, 12H), 2.08 (s, 3H), 1.65 (s, 9H), 0.61 (s, 3H), 0.58 (s, 3H) ppm.

^{13}C NMR (126 MHz, $CDCl_3$): δ 168.7, 165.3, 154.4, 148.8, 142.8, 141.3, 136.3, 132.8, 131.3, 129.2, 127.2, 126.8, 121.1, 114.1, 81.8, 41.0, 28.3, 19.4, -0.8, -1.2 ppm.

HRMS (ESI) calcd. for $C_{31}H_{39}N_2O_2Si$ $[M]^+$: 499.2786; found, 499.2777.

General procedure for the synthesis of amino acids. Compound **4** (35 mg, 0.065 mmol) was dissolved in TFA (1 mL) and CH_2Cl_2 (1 mL), then the solution was evaporated and dissolved in anhydrous DMF (1 mL). DIPEA (80 µL) and PyBOP (135 mg, 0.26 mmol) were added to the solution. The mixture was stirred at r.t. for 2 hours, then the protected amino acid (0.65 mmol, 10 eq.) in anhydrous DMF (1 mL) and DIPEA (113 µL, 0.65 mmol) was added to the reaction mixture. The reaction was stirred at r.t. overnight, concentrated and purified by reversed-phase preparative HPLC to obtain the corresponding amino acids as blue solids.

S)-N-(10-(4-((5-((tert-butoxycarbonyl)amino)-6-methoxy-6-oxohexyl)carbamoyl)-2-methylphenyl)-7-(dimethylamino)-5,5-dimethyldibenzo[b,e]silin-3(5H)-ylidene)-N-methylmethanaminium 2,2,2-trifluoroacetate (5)

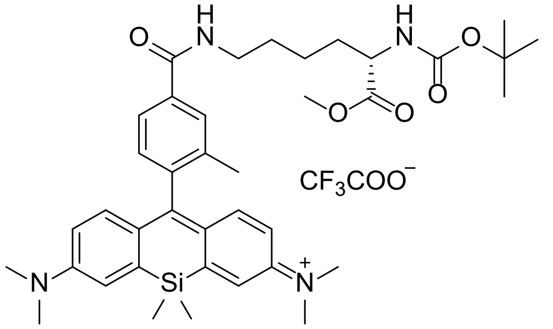

Following the general procedure, the reaction was run using Boc-Lys-OMe HCl (169 mg, 0.65 mmol, 10 eq). The mixture was purified by preparative HPLC (0.1% TFA acetonitrile/water). After lyophilisation, the Boc-protected amino acid **5** was obtained as a blue solid (40 mg, 77%).

^{1}H NMR (500 MHz, $CDCl_3$) δ 7.84 (s, 1H), 7.80 (d, *J* = 8.5 Hz, 1H), 7.31 (s, 1H), 7.15 – 7.12 (m, 3H), 7.08 (d, *J* = 9.7 Hz, 2H), 6.61 (dd, *J* = 9.7, 2.8 Hz, 2H), 5.19 (d, *J* = 8.3 Hz, 1H), 4.29 (q, *J* = 7.4 Hz, 1H), 3.74 (s, 3H), 3.50 (q, *J* = 6.7 Hz, 2H), 3.34 (s, 12H), 2.06 (s, 3H), 1.86 (s, 1H), 1.77 – 1.61 (m, 3H), 1.52 – 1.46 (m, 2H), 1.44 (s, 9H), 0.59 (s, 3H), 0.57 (s, 3H) ppm.

^{13}C NMR (126 MHz, $CDCl_3$): δ 173.5, 170.0, 167.8, 155.7, 154.3, 148.6, 141.9, 141.1, 136.2, 136.0, 129.4, 129.2, 127.5, 124.9, 124.6, 120.7, 114.3, 79.9, 53.5, 52.4, 40.9, 39.8, 32.4, 29.1, 28.4, 22.8, 19.4, -0.8, -1.1 ppm.

HRMS (ESI) calcd. for $C_{39}H_{53}N_4O_5Si$ $[M]^+$: 685.3791; found, 685.3768.

(*S*)-*N*-(10-(4-((4-(2-((*tert*-butoxycarbonyl)amino)-3-methoxy-3-oxopropyl)phenyl)carbamoyl)-2-methylphenyl)-7-(dimethylamino)-5,5-dimethyldibenzo[*b*,*e*]silin-3(5*H*)-ylidene)-*N*-methylmethanaminium 2,2,2-trifluoroacetate (6)

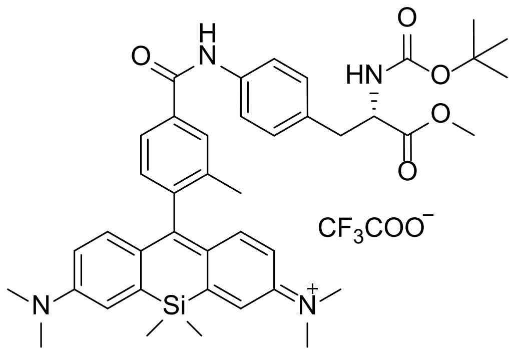

Following the general procedure, the reaction was run using Boc-Phe(4-NH_2)-OMe (191 mg, 0.65 mmol, 10 eq). The mixture was purified by preparative HPLC (0.1% TFA acetonitrile/water). After lyophilisation, the Boc-protected amino acid **6** was obtained as a blue solid (45 mg, 82%).

^{1}H NMR (500 MHz, $CDCl_3$): δ 9.39 (s, 1H), 7.91 (s, 1H), 7.88 (d, *J* = 8.1 Hz, 1H), 7.78 (d, *J* = 8.6 Hz, 2H), 7.17 (d, *J* = 8.1 Hz, 1H), 7.13 – 7.08 (m, 6H), 6.64 (dd, *J* = 9.6, 2.8 Hz, 2H), 4.99 (d, *J* = 8.2 Hz, 1H), 4.57 (q, *J* = 6.5 Hz, 1H), 3.72 (s, 3H), 3.33 (s, 12H), 3.08 (d, *J* = 7.8 Hz, 2H), 2.06 (s, 3H), 1.44 (s, 9H), 0.59 (s, 3H), 0.57 (s, 3H) ppm.

^{13}C NMR (126 MHz, $CDCl_3$): δ172.4, 170.1, 166.4, 154.4, 148.5, 142.0, 141.2, 137.9, 136.7, 136.3, 131.8, 129.8, 129.6, 129.2, 127.5, 124.9, 120.8, 120.6, 114.4, 80.1, 54.6, 52.3, 40.9, 37.8, 28.5, 19.4, -0.7, -1.0 ppm.

HRMS (ESI) calcd. for $C_{42}H_{51}N_4O_5Si$ $[M]^+$: 719.3623; found, 719.3612.

(*S*)-*N*-(10-(4-((2-((*tert*-butoxycarbonyl)amino)-3-methoxy-3-oxopropyl)carbamoyl)-2-methylphenyl)-7-(dimethylamino)-5,5-dimethyldibenzo[*b*,*e*]silin-3(5*H*)-ylidene)-*N*-methylmethanaminium 2,2,2-trifluoroacetate (7)

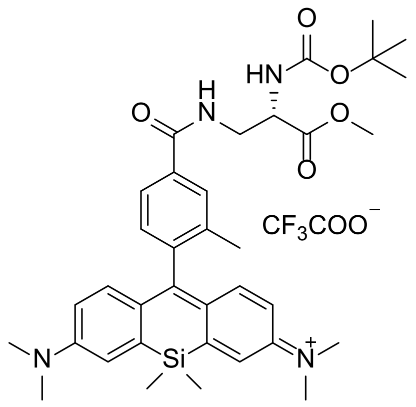

Following the general procedure, the reaction was run using Boc-Dap-OMe HCl (166 mg, 0.65 mmol, 10 eq). The mixture was purified by preparative HPLC (0.1% TFA acetonitrile/water). After lyophilisation, the Boc-protected amino acid **7** was obtained as a blue solid (39 mg, 80%).

^{1}H NMR (500 MHz, $CDCl_3$): δ 7.84 (s, 2H), 7.79 (d, *J* = 7.9 Hz, 1H), 7.17 – 7.12 (m, 3H), 7.05 (d, *J* = 9.7 Hz, 2H), 6.61 (dd, *J* = 9.7, 2.7 Hz, 2H), 6.01 (d, *J* = 7.9 Hz, 1H), 4.53 (s, 1H), 3.90 (s, 2H), 3.81 (s, 3H), 3.34 (s, 12H), 2.05 (s, 3H), 1.45 (s, 9H), 0.59 (s, 3H), 0.57 (s, 3H) ppm.

^{13}C NMR (126 MHz, $CDCl_3$): δ 171.2, 169.6, 168.2, 160.5, 160.2, 156.4, 154.3, 148.6, 141.8, 141.4, 136.3, 135.2, 129.5, 129.2, 127.4, 124.8, 120.8, 117.3, 115.0, 114.2, 80.3, 54.2, 52.8, 42.7, 41.0, 28.4, 19.4, -0.8, -1.1 ppm.

HRMS (ESI) calcd. for $C_{36}H_{47}N_4O_5Si$ $[M]^+$: 643.3310; found, 643.3299.

(*S*)-*N*-(10-(4-((4-(*tert*-butoxy)-3-((*tert*-butoxycarbonyl)amino)-4-oxobutyl)carbamoyl)-2-methylphenyl)-7-(dimethylamino)-5,5-dimethyldibenzo[*b*,*e*]silin-3(5*H*)-ylidene)-*N*-methylmethanaminium 2,2,2-trifluoroacetate (8)

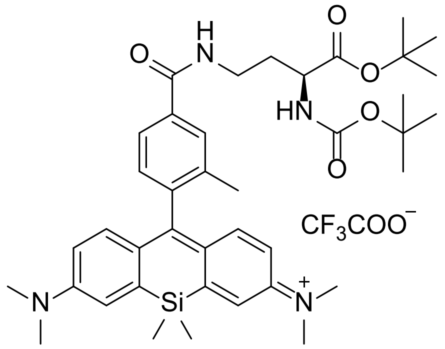

Following the general procedure, the reaction was run using Boc-Dab-OtBu HCl (169 mg, 0.65 mmol, 10 eq). The mixture was purified by preparative HPLC (0.1% TFA acetonitrile/water). After lyophilisation, the Boc-protected amino acid **8** was obtained as a blue solid (34 mg, 64%).

^{1}H NMR (500 MHz, $CDCl_3$): δ 7.92 (s, 2H), 7.86 (d, *J* = 7.8 Hz, 1H), 7.21 – 7.16 (m, 3H), 7.05 (d, *J* = 9.5 Hz, 2H), 6.59 (dd, *J* = 9.5, 2.6 Hz, 2H), 5.41 (d, *J* = 8.0 Hz, 1H), 4.29 (s, 1H), 3.35 (s, 12H), 3.21 (s, 1H), 2.23 (s, 1H), 2.09 (s, 3H), 1.72 (s, 1H), 1.48 (s, 18H), 1.42 (s, 1H), 0.60 (s, 3H), 0.58 (s, 3H) ppm.

^{13}C NMR (126 MHz, $CDCl_3$): δ 171.7, 154.3, 148.8, 141.6, 136.4, 135.4, 129.4, 129.3, 127.4, 124.5, 121.0, 114.1, 82.8, 80.6, 51.8, 41.0, 36.1, 33.6, 28.4, 28.1, 19.5, -0.9, -1.1 ppm.

HRMS (ESI) calcd. for $C_{40}H_{55}N_4O_5Si$ $[M]^+$: 699.3936; found, 699.3907.

(*S*)-*N*-(10-(4-((5-(*tert*-butoxy)-4-((*tert*-butoxycarbonyl)amino)-5-oxopentyl)carbamoyl)-2-methylphenyl)-7-(dimethylamino)-5,5-dimethyldibenzo[*b*,*e*]silin-3(5*H*)-ylidene)-*N*-methylmethanaminium 2,2,2-trifluoroacetate (9)

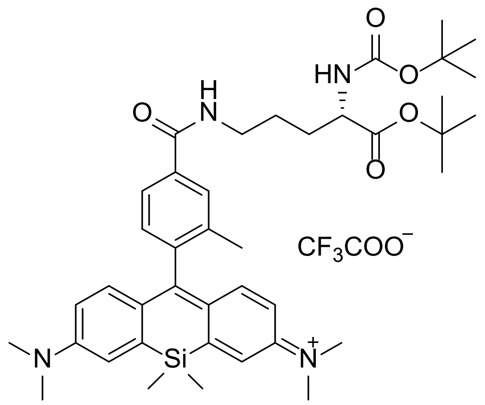

Following the general procedure, the reaction was run using Boc-Orn-OtBu HCl (188 mg, 0.65 mmol, 10 eq). The mixture was purified by preparative HPLC (0.1% TFA acetonitrile/water). After lyophilisation, the Boc-protected amino acid **9** was obtained as a blue solid (35 mg, 65%).

^{1}H NMR (500 MHz, $CDCl_3$): δ 7.89 (s, 1H), 7.85 (d, *J* = 7.7 Hz, 1H), 7.76 (s, 1H), 7.16 – 7.12 (m, 3H), 7.08 (d, *J* = 9.6 Hz, 2H), 6.61 (dd, *J* = 9.6, 2.8 Hz, 2H), 5.29 (d, *J* = 7.7 Hz, 1H), 4.26 – 4.13 (m, 1H), 3.66 – 3.43 (m, 2H), 3.34 (s, 12H), 2.05 (s, 3H), 1.90 (s, 1H), 1.78 (s, 3H), 1.47 (s, 9H), 1.44 (s, 9H), 0.59 (s, 3H), 0.57 (s, 3H) ppm.

^{13}C NMR (126 MHz, $CDCl_3$): δ 171.9, 170.0, 167.6, 155.8, 154.3, 148.6, 141.9, 141.0, 136.1, 136.0, 129.5, 129.1, 127.5, 124.7, 120.7, 114.2, 82.1, 79.7, 53.8, 40.9, 39.7, 30.7, 28.4, 28.1, 25.3, 19.4, -0.8, -1.1 ppm.

HRMS (ESI) calcd. for $C_{41}H_{57}N_5O_5Si$ $[M]^+$: 713.4092; found, 713.4083.

(*S*)-*N*-(10-(4-((5-acetamido-6-methoxy-6-oxohexyl)carbamoyl)-2-methylphenyl)-7-(dimethylamino)-5,5-dimethyldibenzo[*b*,*e*]silin-3(5*H*)-ylidene)-*N*-methylmethanaminium 2,2,2-trifluoroacetate (10)

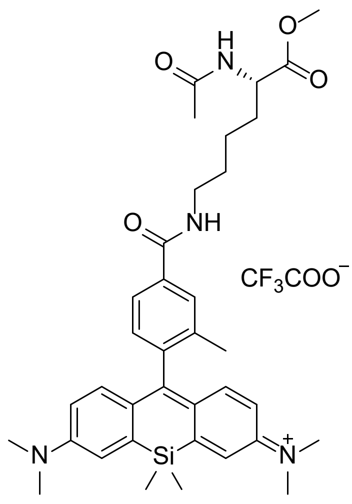

Following the general procedure, the reaction was run using Nα-Ac-Lys-OMe HCl (155 mg, 0.65 mmol, 10 eq). The mixture was purified by preparative HPLC (0.1% TFA acetonitrile/water). After lyophilisation, the Boc-protected amino acid **10** was obtained as a blue solid (33 mg, 70%).

^{1}H NMR (500 MHz, $CDCl_3$): δ 7.89 (s, 1H), 7.85 (d, *J* = 7.5 Hz, 1H), 7.78 (s, 1H), 7.14 – 7.09 (m, 4H), 7.04 – 6.97 (m, 1H), 6.63 (dd, *J* = 9.6, 2.8 Hz, 2H), 4.59 – 4.48 (m, 1H), 3.73 (s, 3H), 3.60 – 3.41 (m, 2H), 3.34 (s, 12H), 2.05 (s, 3H), 2.04 (s, 3H), 1.95 – 1.70 (m, 3H), 1.70 – 1.59 (m, 1H), 1.58 – 1.40 (m, 2H), 0.59 (s, 3H), 0.57 (s, 3H) ppm.

^{13}C NMR (126 MHz, $CDCl_3$): δ 173.0, 170.5, 168.1, 154.4, 148.5, 142.1, 140.9, 136.0, 136.0, 129.6, 129.0, 127.5, 124.8, 120.5, 114.3, 52.5, 52.4, 40.9, 39.4, 31.4, 28.6, 22.8, 22.5, 19.3, -0.7, -1.0 ppm.

HRMS (ESI) calcd. for $C_{36}H_{47}N_4O_4Si$ $[M]^+$: 627.3361; found, 627.3369.

_N_-(10-(4-(((_S_)-5-((((1_R_,8_S_,9_s_)-bicyclo[6.1.0]non-4-yn-9-ylmethoxy)carbonyl)amino)-5-carboxypentyl)carbamoyl)-2-methylphenyl)-7-(dimethylamino)-5,5-dimethyldibenzo[_b_,_e_]silin-3(5_H_)-ylidene)-_N_-methylmethanaminium 2,2,2-trifluoroacetate (11)

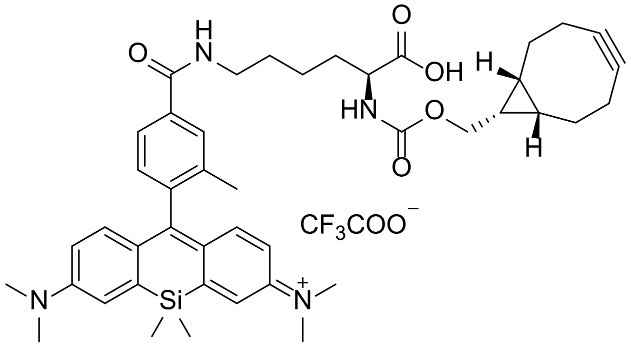

A mixture of compound **5** (40 mg, 0.050 mmol) in 0.25 M NaOH/MeOH (1.5 mL) was stirred at r.t. for 4 hours. The reaction mixture was neutralised with AcOH until the solution turned blue, diluted with H_2O (10 mL) and lyophilised. The obtained blue solid was dissolved in CH_2Cl_2 (5 mL) and transferred into a tube, followed by the addition of trifluoroacetic acid (5 mL). The mixture was stirred at r.t. for 30 minutes. The solution was concentrated under reduced pressure and dissolved in anhydrous DMF (2 mL). A mixture of BCN-succinimidyl ester (44 mg, 0.15 mmol) and DIPEA (87 µL, 0.50 mmol) in anhydrous DMF (1 mL) was added to the reaction mixture and stirred at r.t. for 30 minutes. The mixture was purified by preparative HPLC (0.1% TFA acetonitrile/water). After lyophilisation, compound **11** was obtained as a blue solid (13 mg, 30%).

^{1}H NMR (500 MHz, $CDCl_3$) δ 7.84 (s, 1H), 7.81 (d, J = 7.7 Hz, 2H), 7.13 (d, J = 7.7 Hz, 1H),

7.10 – 7.07 (m, 3H), 6.65 (dd, *J* = 9.4, 2.8 Hz, 2H), 5.80 – 5.46 (m, 1H), 4.49 – 4.42 (m, 2H), 4.14 (d, *J* = 7.7 Hz, 1H), 3.58 – 3.01 (m, 12H), 2.32 – 2.16 (m, 9H), 2.04 (s, 3H), 1.74 – 1.66 (m, 2H), 1.61 – 1.46 (m, 6H), 1.39 – 1.31 (m, 1H), 1.08 – 1.02 (m, 1H), 0.96 – 0.89 (m, 1H), 0.58 (s, 3H), 0.56 (s, 3H) ppm.

^{13}C NMR (126 MHz, $CDCl_3$) δ 174.3, 171.8, 170.3, 168.8, 156.6, 154.4, 151.8, 148.4, 142.1, 141.2, 136.2, 135.5, 129.6, 129.1, 127.5, 124.9, 120.5, 119.3, 118.5, 117.0, 114.7, 114.5, 98.8, 70.5, 67.1, 63.1, 60.2, 53.9, 40.9, 29.1, 28.4, 25.6, 24.4, 22.1, 21.6, 21.5, 20.8, 20.3, 19.3, 17.3, -0.7, -1.0 ppm.

HRMS (ESI) calcd. for $C_{44}H_{55}N_4O_5Si$ $[M]^+$: 747.3936; found, 747.3941**.**

(4-(((((2*S*,3*S*,4*S*,6*R*)-3-hydroxy-2-methyl-6-(((1*S*,3*S*)-3,5,12-trihydroxy-3-(2-hydroxyacetyl)-10-methoxy-6,11-dioxo-1,2,3,4,6,11-hexahydrotetracen-1-yl)oxy)tetrahydro-2*H*-pyran-4-yl)carbamoyl)oxy)methyl)phenyl)boronic acid (*caged doxorubicin*)

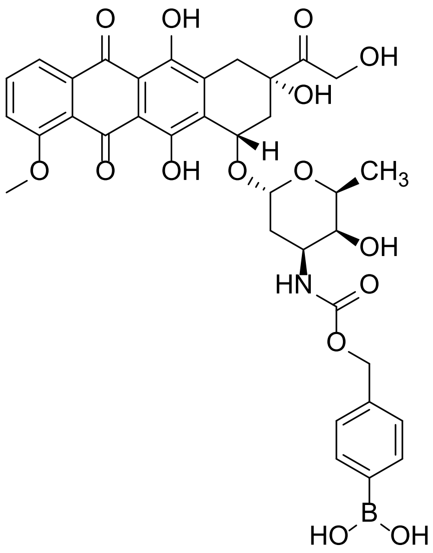

Caged doxorubicin was prepared following reported procedures.[2] A mixture of 4-nitrophenyl (4-(4,4,5,5-tetramethyl-1,3,2-dioxaborolan-2-yl)benzyl) carbonate (25 mg, 0.063 mmol) and doxorubicin hydrochloride (45 mg, 0.078 mmol) in DMF:H_2O (2 mL, 1:1) was stirred at r.t. for 2 hours. The crude material was purified by preparative HPLC (0.1% TFA acetonitrile/water). After lyophilisation, the compound was isolated as a red solid (14 mg, 31%).

HRMS (ESI) calcd. for $C_{35}H_{36}BNO_{15}Na$ $[M+Na]^+$: 744.2070; found, 744.2058**.**

pdCpA coupling. The Boc-protected amino acids **5-9** (12 mg) were individually dissolved in 0.25 M NaOH in MeOH (1.5 mL) to hydrolyze the esters. The reactions were monitored by HPLC and, upon completion the mixtures were neutralised with a few drops of AcOH until the solutions turned blue. The resulting solutions were diluted with H_2O (3 mL) and lyophilised. Next, *N*,*N*'-carbonyldiimidazole (7 mg, 0.043 mmol) was dissolved in anhydrous DMF (60 µL) and added to the different hydrolysed amino acids. The reaction mixtures were incubated at r.t. for 3 minutes, followed by the addition of pdCpA (2.5 mg dissolved in 55 µL of water, pH 8.3). Subsequently, to remove unreacted amino acids, the solutions were divided into six 1.5 mL microcentrifuge tubes and diluted with 1.2 mL of anhydrous THF. The solutions were centrifuged at maximum speed for 5 min, and the surfactants were removed. The pellets were dried under N_2 and dissolved in a mixture of H_2O:ACN (1:1), followed by purification using semi-preparative HPLC. Finally, the Boc-protected AA-pdCpA conjugates were transferred to a 1.5 mL microcentrifuge tube, treated with cold TFA (1.0 mL) and stirred on ice for 1 h. TFA was evaporated by using N_2 and the products were precipitated in 1 mL cold Et_2O. The solutions were centrifuged at maximum speed for 5 min, and the resulting pellets were dried at r.t. for 10 min. Subsequently, dried pellets were dissolved in DMSO (final concentration 3 mM) for tRNA ligation or stored at -80°C for later use.

Spectroscopic measurements. Spectroscopic data were recorded on a Synergy HT

spectrophotometer (Biotek) and data analysis was performed using GraphPad Prism 10.0. Stock solutions were prepared in DMSO and diluted with the indicated solvents. Spectra were recorded at r.t.

1,3-Diphenylisobenzofuran (DPBF) assays. All compounds (50 μM) were dissolved in EtOH and incubated with DPBF (300 μM), followed by irradiation with a ThorLabs M625L3 (0.40 mW cm^{-2}) single-color mounted LED for 4 minutes. Absorbance at 410 nm was recorded before and after illumination, and percentages of decrease in absorption for DPBF were calculated.

Singlet oxygen quantum yields. The absorbance value of solutions containing DPBF (300 μM) and the compounds (20 μM) in EtOH were measured at 410 nm and 620 nm prior to illumination with an LED light source (0.40 mW cm^{-2}, 4 minutes). After illumination, absorbance measurements at 410 nm were taken at different time points. The rates of singlet oxygen production (k) were determined by plotting the change in DPBF absorbance at 410 nm over time, using the following equation for calculation.[3]

$$k = \frac{\ln(DPBF\,Abs_t)}{\ln(DPBF\,Abs_0)}\ \ln\,(PSAbs_{exc})$$

Relative singlet oxygen quantum yields (Φ_{Δ_PS}) were determined using methylene blue as standard. The rates of singlet oxygen production (k) were calculated from the experimental

data, and these values were used to determine the singlet oxygen quantum yields (Φ_{Δ_PS}) for all compounds.

$$\Phi_{\Delta PS} = \Phi_{\Delta ref} \frac{k_{PS}}{k_{ref}}$$

tRNA ligation and analysis. Truncated McTrp tRNA (300 µg, lacking the CA terminal motif) was dissolved in 375 µL of 10 mM HEPES buffer containing 2.5 mM $MgCl_2$. To fold the tRNA, the solution was heated to 95°C for 3 minutes and incubated at r.t. for 20 minutes. In parallel, the ligation master mix was prepared by combining 29 µL of water, 8.3 µL of 10× T4 RNA ligase buffer, 1 µL of 10 mM ATP, and 5 µL of T4 RNA ligase 1 (NEB). The reaction was assembled at 4°C in a 1.5 mL microcentrifuge tube by adding 8 µL of pdCpA-**5**/**9** (3 mM), 42 µL of the ligation master mix, and 30 µL folded tRNA solution. The mixture was incubated on ice for 1 h. The labelled tRNA was extracted with 500 µL phenol:$CHCl_3$ (5:1) containing 0.1 M sodium acetate (pH 5.2) and precipitated in EtOH overnight at -20°C. The next day, the pellet was washed with 500 µL of 70% EtOH and the pellet was air dried.

Cloning of proteins into NEB Express® vectors. Plasmids containing the wild-type PD-L1 and IL-33 genes were constructed by amplifying the NEBExpress® backbone (full plasmid supplied with NEBexpress IVTT kits) using a 25-cycle PCR with Q5® High-Fidelity 2× Master Mix. The circular template in the reaction mixture was digested with DpnI, and the backbone

was purified using the Monarch® PCR & DNA Cleanup Kit. Plasmids were then constructed and amplified by assembling the linear backbone with g-blocks (IDT) using the NEBuilder® HiFi DNA Assembly Kit (2 μL reactions), followed by cloning into NEB® 5-alpha Competent *E. coli*. All primers and g-blocks were designed according to the manufacturer's instructions, and the sequences were verified through whole plasmid sequencing (plasmidsaurus).

Site-directed mutagenesis. The NEB Express® Control DHFR-His plasmid and the above IL-33 and PD-L1 plasmids, were subjected to site-directed mutagenesis using the Q5® Site-Directed Mutagenesis Kit. Primers were designed following the manufacturer's instructions to insert a TAG codon at position 2. Mutated plasmids were then amplified by cloning into NEB® 5-alpha Competent *E. coli* and sequences were verified via whole plasmid sequencing (plasmidsaurus).

***In vitro* transcription and translation (IVTT).** IVTT reactions were carried out using the NEB Express cell-free *E. coli* Protein Synthesis System kit following the manufacturer's instructions. The kit was supplemented with the DNA template for the desired protein (20 ng μL^{-1}), SiRAA -tRNA (8 μM), 1.5 units μL^{-1} RNase Inhibitor Murine and Api 137 (50 μg mL^{-1}). Reactions (5-250 μL) were incubated in 1.5 mL microcentrifuge tubes or 0.2 mL PCR tubes at 37°C for 4 hours. For analysis, 2 μL of the translation mixture was run alongside 2 μL of

PageRuler™ Prestained Protein Ladder on 4-12% NuPAGE Bis-Tris protein gels, following the manufacturer's instructions. The gels were scanned for in-gel fluorescence using the ChemiDoc MP Gel Doc (BioRad), then transferred to a nitrocellulose membrane with the iBlot™ 2 Gel Transfer Device for Western Blot analysis. The membranes were blocked in 10% w/v non-fat dry milk in PBS supplemented with 0.1% Tween-20, for 1 hour at r.t. Subsequently, primary antibodies were diluted in 1% w/v non-fat dry milk in PBS-T and incubated with the membrane overnight at 4°C. The following day, membranes were washed with 25 mL of PBS-T (10 min × 3), then incubated for 1 hour at r.t. with an HRP-conjugated secondary antibody. After three additional washes with PBS-T, the membranes were treated with ECL and scanned using a C-DiGit® Blot Scanner. Images were analysed using ImageLab software.

PD-L1-pAzF protein expression. SHuffle® T7 Competent cells were thawed and transformed with 50 ng of each plasmid, pEVOL-pAzF (a gift from Peter Schultz, Addgene plasmid #31186), and pNEB-PDL1-TAG2, following the manufacturer's protocol. The next day, a single colony was selected to inoculate 10 mL of LB broth supplemented with ampicillin (100 µg mL^{-1}) and chloramphenicol (50 µg mL^{-1}). The culture was incubated overnight at 30°C with constant stirring. The following morning, the OD_{600} of the overnight culture was measured and used to inoculate 40 mL of fresh LB broth containing both antibiotics, adjusting the initial

OD_{600} to 0.1–0.2. The suspension was incubated at 30°C with constant shaking for 3–4 hours until the OD_{600} reached 0.5–0.6. A solution of 4-azido-L-phenylalanine in 1 M NaOH was then added to a final concentration of 2 mM, and protein expression was induced by the addition of 1 mM IPTG. The culture was incubated overnight at 30°C with continuous shaking. The next day, the OD_{600} was recorded, and the cells were harvested by centrifugation at 4,000 × g for 15 minutes at 4°C. The supernatant was discarded, and the pellet was resuspended in 3.4 mL of NEB lysis buffer reagent. T4 lysozyme (1 µL mL^{-1} lysis buffer), micrococcal nuclease (1 µL mL^{-1} lysis buffer), and 1 M $CaCl_2$ (1 µL mL^{-1} lysis buffer) were added, and the suspension was stirred at room temperature for 20 minutes. Following lysis, the insoluble fraction was collected by centrifugation at 17,000 × g for 15 minutes at 4°C and stored at −20°C pending purification.

Protein purification and refolding. The insoluble fraction was resuspended in 1 mL of denaturing buffer (6 M guanidine hydrochloride, 300 mM NaCl, 50 mM sodium phosphate, 10 mM imidazole, pH 7.4) and added to 1 mL of pre-equilibrated HisPur™ Cobalt Resin. The resin was washed three times with 1 mL of denaturing buffer. Protein elution was carried out by incubating the resin sequentially with three 1 mL aliquots of denaturing elution buffer (6 M guanidine hydrochloride, 300 mM NaCl, 50 mM sodium phosphate, 300 mM imidazole, pH 7.4). Eluted fractions containing protein were pooled and added dropwise to pre-chilled refolding buffer (880 mM L-arginine, 55 mM Tris-HCl, 21 mM NaCl, 0.88 mM KCl, pH 8.3) to

achieve a 20-fold dilution. The solution was stirred continuously at 4°C overnight to facilitate protein refolding. The following day, the protein solution was concentrated using Amicon Ultra-15 centrifugal filters (3 kDa cutoff, Merck). Protein purity and identity were evaluated via SDS-PAGE and mass spectrometry.

Labelling of PD-L1-pAzF protein with compound 11. PD-L1-pAzF (15 μM) in refolding buffer was incubated with 5 equivalents of compound **11** dissolved in DMSO (final DMSO concentration: 2%). The reaction was stirred overnight at 4°C in the dark. The following day, the reaction mixture was purified using Zeba Spin Desalting columns (2 mL, 7 kDa MWCO) pre-equilibrated with PBS, following the manufacturer's instructions. The concentration and degree of labelling (DoL) of the purified protein were determined by measuring absorbance at 280 nm and 650 nm using a Nanodrop One spectrophotometer (Thermo Fisher Scientific). The DoL was calculated to be approximately 1. The labelled protein was further analysed via SDS-PAGE using a 4–12% Bis-Tris polyacrylamide gel. Fluorescence of the labelling reaction product was detected by scanning the gel in the red channel.

T cell isolation, culture and activation. Whole blood was collected from healthy volunteers following informed consent and ethical approval (21-EMREC-041) at the University of Edinburgh. T cells were isolated from the blood by immunomagnetic negative selection using

easySep™ Direct Human T cell Isolation Kit. T cells were then seeded at 100,000 cells/well on a 96-well flat-bottomed plate and cultured for 72 hours in Dulbecco's Modified Eagle Medium containing 10% fetal bovine serum, 2-mercaptoethanol, L-glutamine and Pen-Strep at 37°C and 5% CO_2. T cells were activated using Dynabeads™ Human T-activator CD3/CD28 for 72 hours (1:1 cell to bead ratio). Beads were separated using an Easysep magnet (Stemcell technologies, Cat no- 18000). Both non-activated and activated T cells were treated with anti-PD1 antibody (Biolegend, clone: EH12.2H7, cat. 329936, dilution 1:100) for 20 min at 4°C followed by washing with PBS. T cells were resuspended in FACS buffer (PBS+0.1% BSA) and PD1 expression was determined by flow cytometry. Results were analysed using FCS express 7 (De Novo Software).

Photocatalytic activation of DHR using PD-L1-11 in T cells. T cells were isolated and activated as described above. Both activated and non-activated T cells (100,000 cells per condition) were incubated with **PD-L1-11** (5 µM) for 30 minutes at 4°C in the dark. Following incubation, cells were washed and resuspended in PBS containing DHR (0.5 µM) and Zombie Violet Live/Dead stain (1:200 dilution). The samples were irradiated for 1 hour (640 nm, 10 mW cm^{-2}). Finally, cells were analysed by flow cytometry on an Attune NxT flow cytometer and data processed using FlowJo software and plotted with GraphPad Prism.

Cytotoxicity assays. T cells were treated with Zombie Violet Live/Dead stain (dilution 1:100) for 20 min at 4°C followed by washing with PBS. Cells were resuspended in FACS buffer (PBS+0.1% BSA) and cytotoxicity was determined by flow cytometry. Results were analysed using FCS express 7 (De Novo Software).

3. Supplementary Figures

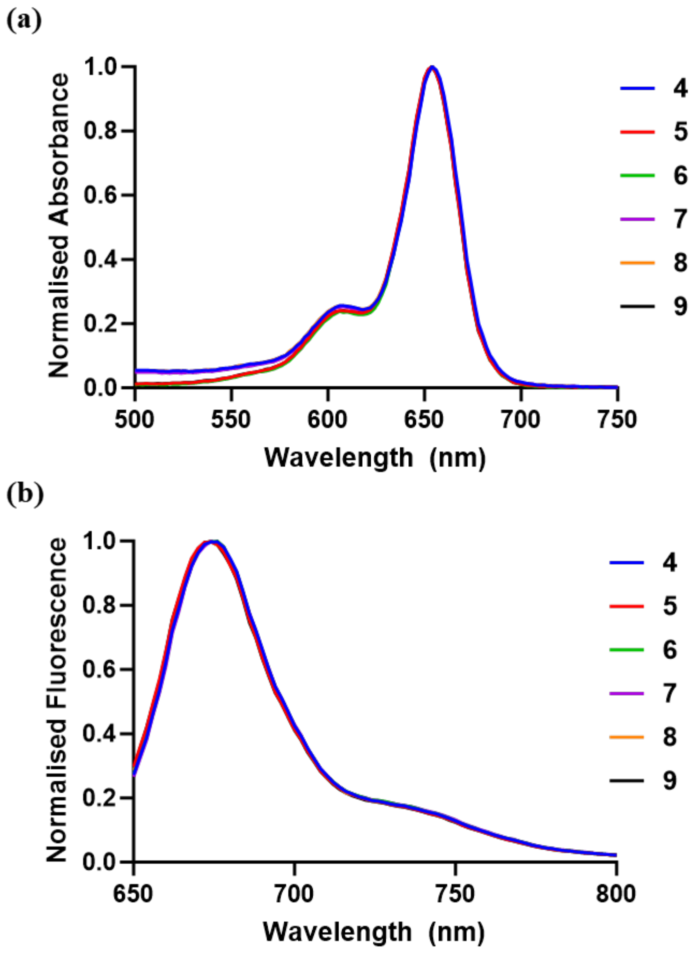

Figure S1. Normalised absorbance and fluorescence emission spectra of compounds 4-9. Representative normalised absorbance (a) and fluorescence spectra (b) of compounds **4-9** (10 µM) in EtOH (n=3). λ_{exc}: 620 nm.

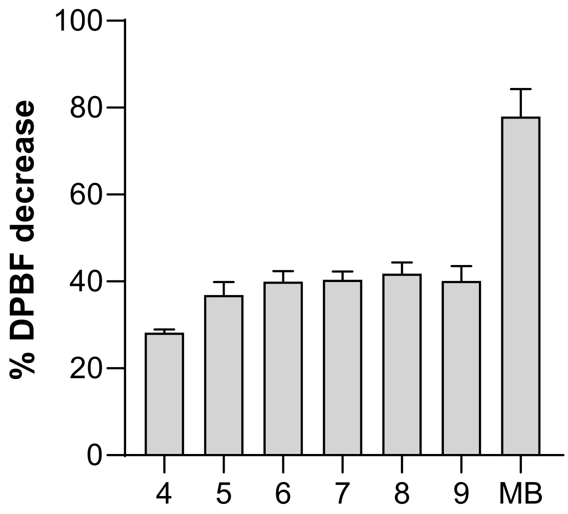

Figure S2. Singlet oxygen generation of compounds 4-9. The percentages of absorbance decrease in DPBF at 410 nm were measured as detailed in the Experimental procedures by incubation of the compounds **4-9** (20 μM) with DPBF (300 μM) in EtOH. Illumination was performed for 4 minutes (0.40 mW cm^{-2}, 640 nm). Methylene blue (MB) was used as a positive control. Values presented as means±SD (n=3).

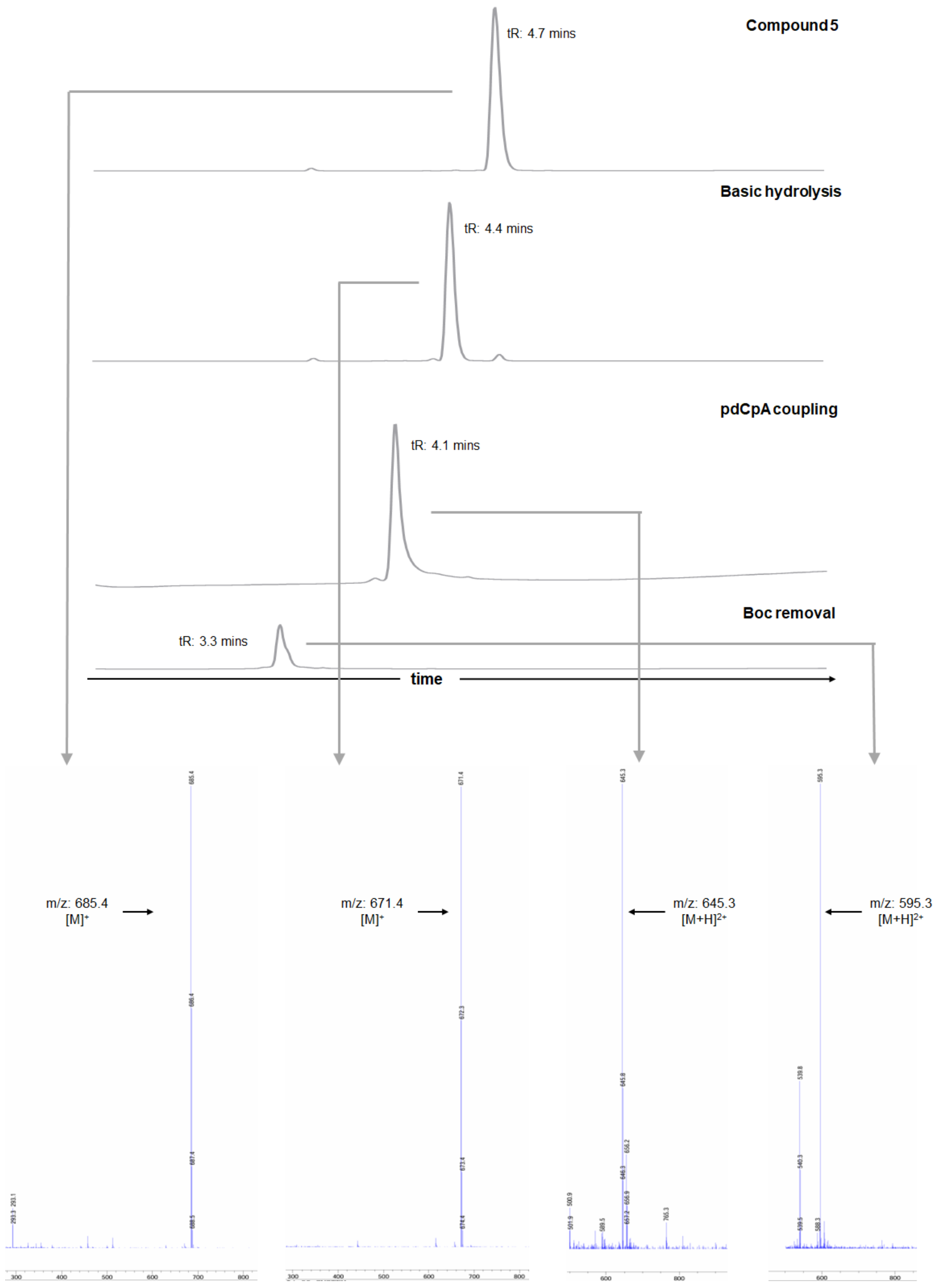

Figure S3. HPLC-MS traces of compound 5 before and after coupling to pdCpA. HPLC traces and corresponding mass data of compound **5** before and after basic hydrolysis, coupling to pdCpA and Boc removal. The pdCpA-**5** conjugate was isolated with purity >90%. UV detection: 650 nm.

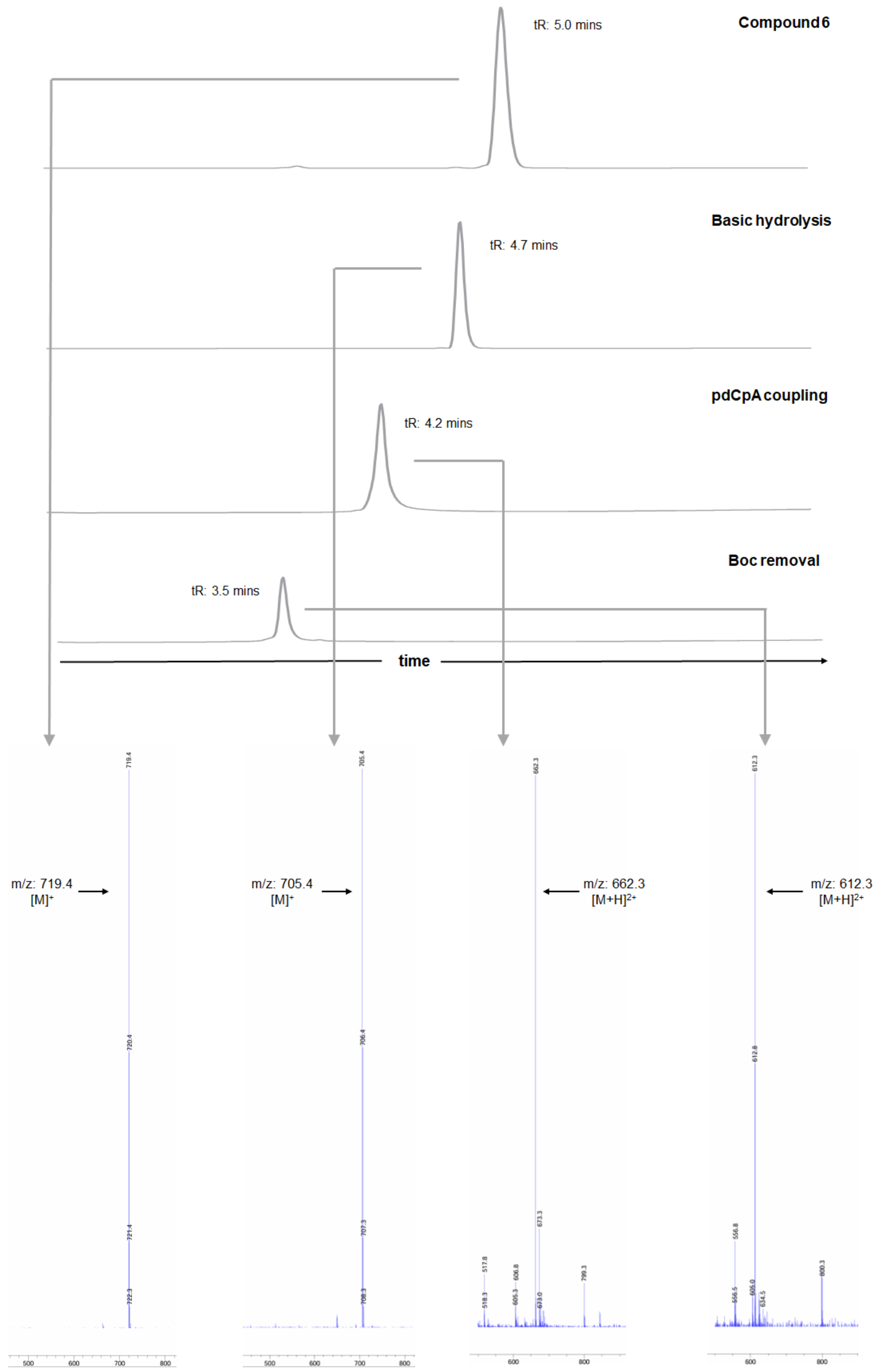

Figure S4. HPLC-MS traces of compound 6 before and after coupling to pdCpA. HPLC traces and corresponding mass data of compound **6** before and after basic hydrolysis, coupling to pdCpA and Boc removal. The pdCpA-**6** conjugate was isolated with purity >90%. UV detection: 650 nm.

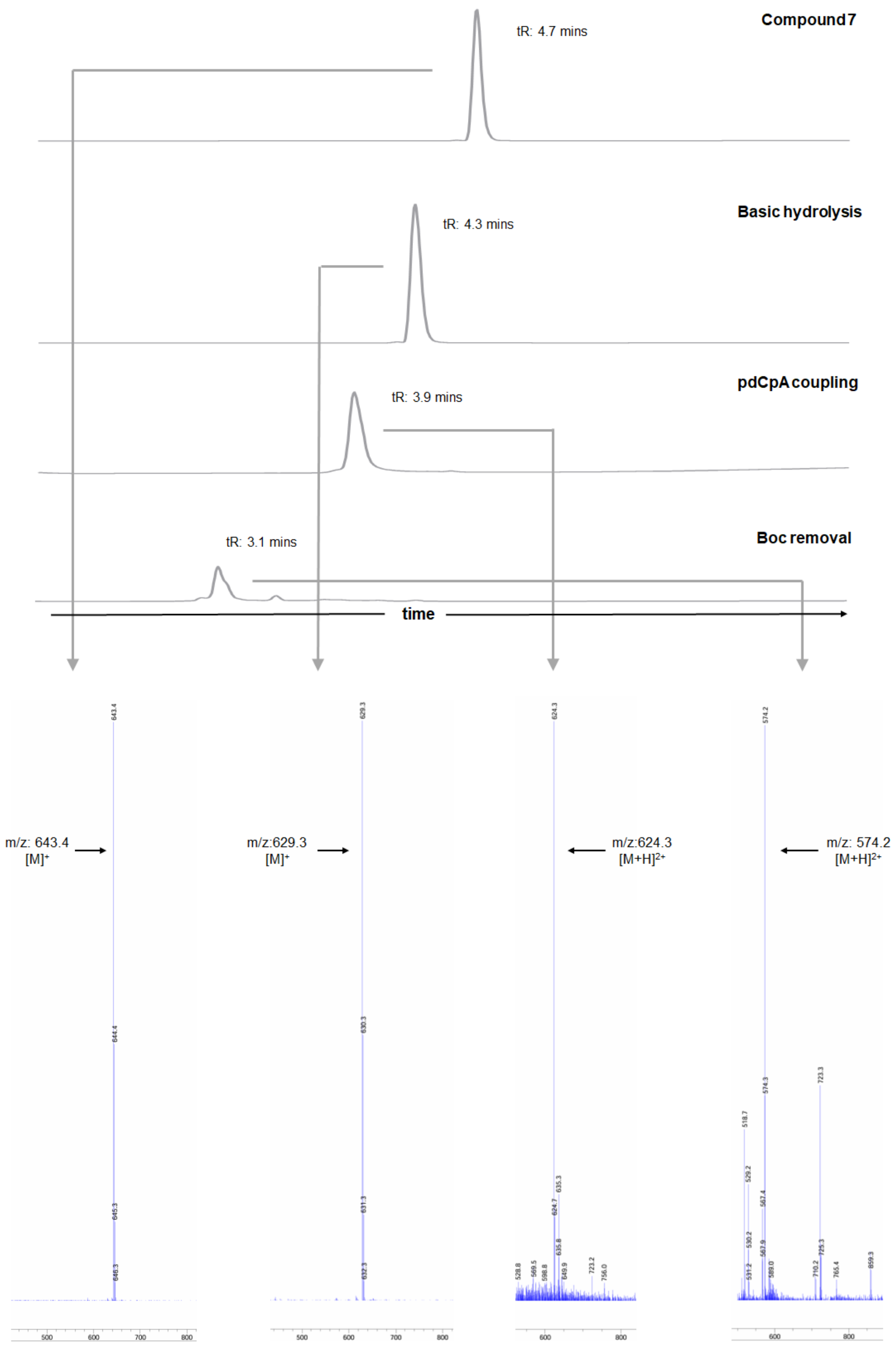

Figure S5. HPLC-MS traces of compound 7 before and after coupling to pdCpA. HPLC traces and corresponding mass data of compound **7** before and after basic hydrolysis, coupling to pdCpA and Boc removal. The pdCpA-**7** conjugate was isolated with purity >90%. UV detection: 650 nm.

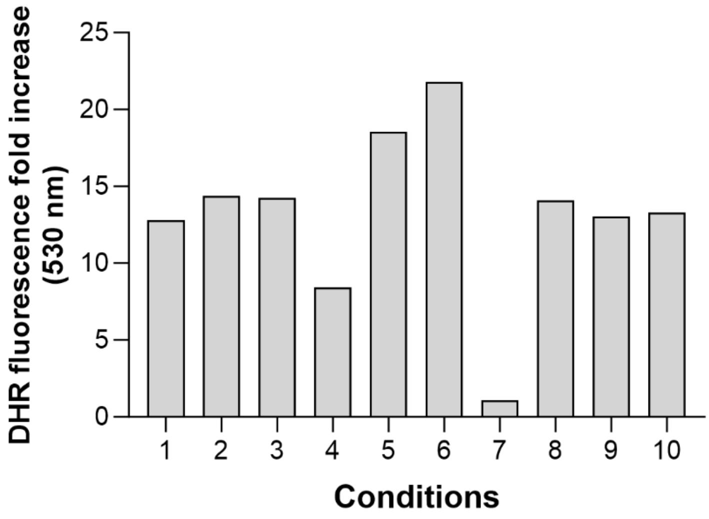

Entry	[5, µM]	[DHR, µM]	Illumination (mW cm^{-2})
1	100	100	7
2	50	100	7
3	25	100	7
4	0	100	7
5	100	50	7
6	100	25	7
7	100	100	-
8	100	100	10
9	100	100	4
10	2 (MB)	100	7

Figure S6. **Fluorescence fold increase of DHR after photocatalysed oxidation using compound 5.** Experiments were performed at the indicated concentrations of compound **5** and DHR in PBS buffer (pH 7.4). The fluorescence emission of all crude mixtures was measured before and after light irradiation (exc: 500 nm, em: 530 nm). DHR: dihydrorhodamine 123.

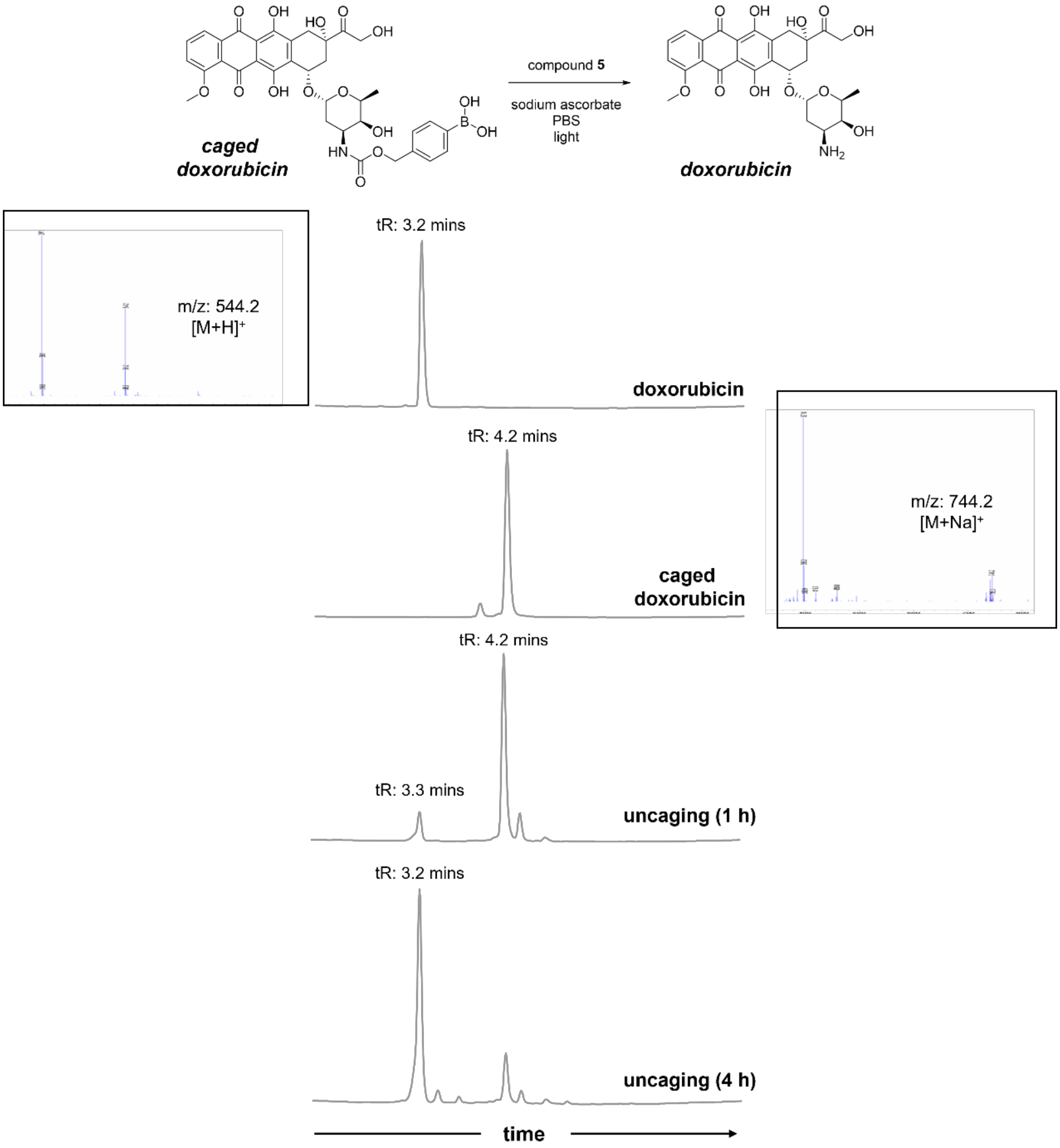

Figure S7. **Deboronative hydroxylation of caged doxorubicin using compound 5.** Chemical reaction of doxorubicin uncaging using the photocatalyst compound **5**. HPLC-MS traces of caged doxorubicin before and after photocatalysis. The reactions were performed after light irradiation (7 mW cm^{-2}, 640 nm, 4 h) using 50 μM compound **5**, 2 mM of sodium ascorbate and 200 μM caged doxorubicin in PBS buffer (pH 7.4).

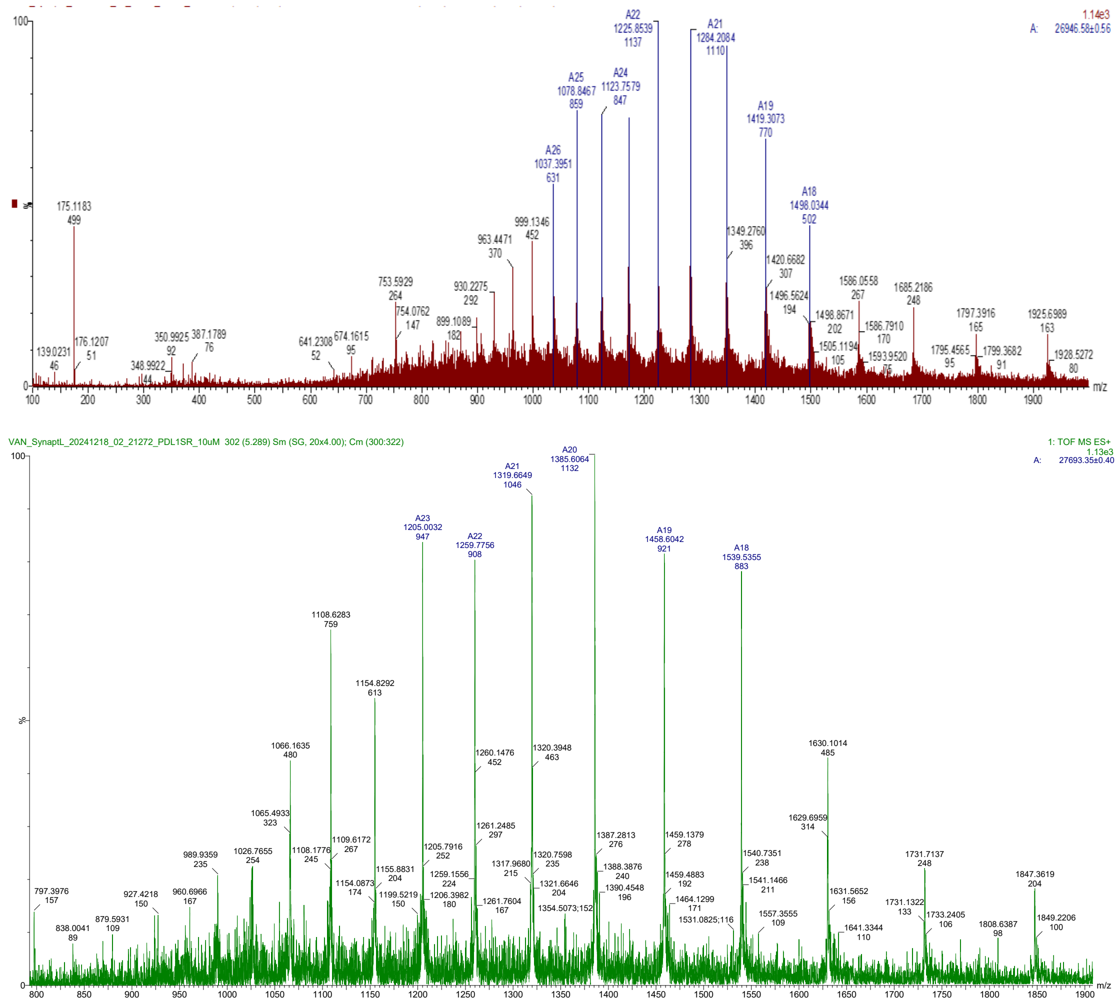

Figure S8. MS spectra for PD-L1 protein analogues. Top panel) PD-L1(azidoPhe) Calculated mass was determined as of 26946 Da. Detected mass was found as of 26946.6±0.6 Da. Bottom panel) **PD-L1-11**. MS s Calculated mass was determined as of 27693 Da. Detected mass was found as of 27693.2±0.1 Da.

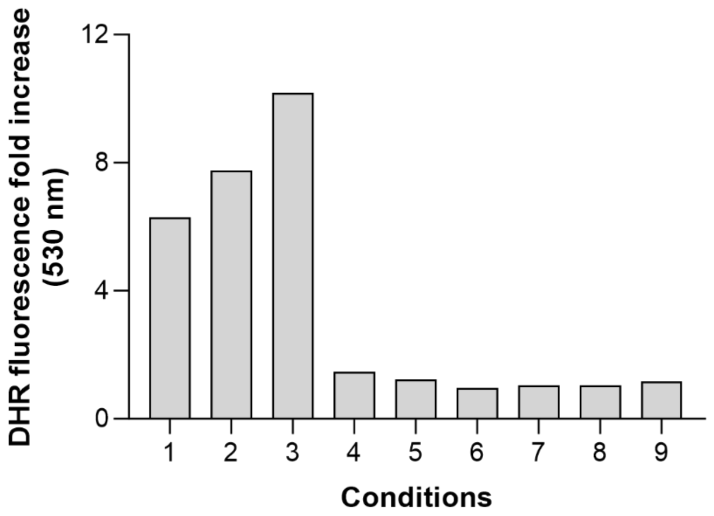

Entry	[PD-L1-11, µM]	[DHR, µM]	Illumination (mW cm^{-2})
1	10	10	10
2	10	5	10
3	10	1	10
4	0	10	10
5	0	5	10
6	0	1	10
7	10	10	-
8	10	5	-
9	10	1	-

Figure S9. Fluorescence fold increase after photocatalysed oxidation of DHR using PD-L1-11 protein. Experiments were performed at the indicated concentrations of **PD-L1-11** and DHR in PBS buffer (pH 7.4). The fluorescence emission of all crude mixtures were recorded before and after light irradiation (exc: 500 nm, em: 530 nm). DHR: dihydrorhodamine 123.

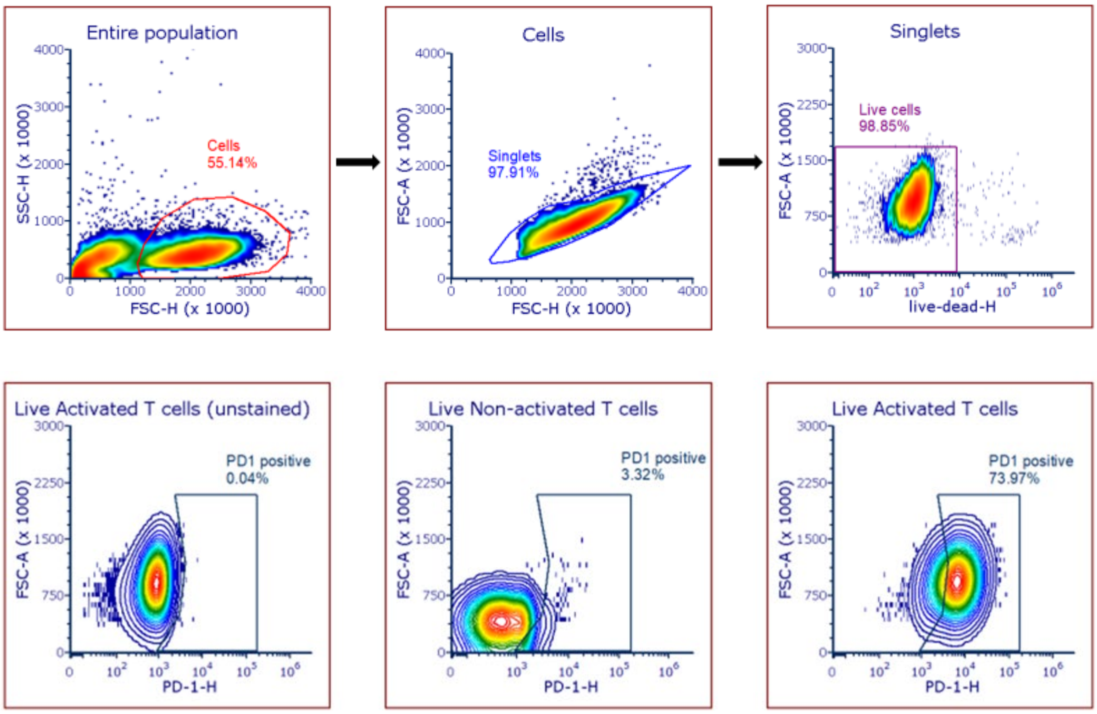

Figure S10. Gating strategy to determine PD-1 expression in stimulated and non-stimulated T cells. The top panel shows the stepwise gating strategy starting from the entire population. Cells were gated first based on the forward and side scattering eliminating all debris and singlets were gated next based on forward scatter area versus height. Live cells were selected from the singlets based on live-dead staining (Zombie Violet Live/Dead stain, 1:100). The bottom panel shows PD-1 positive cells that were gated based on the unstained samples and the quantification was measured as a percentage of cells having positive PD-1 signal. The images were acquired on FCS express 7 (De Novo).

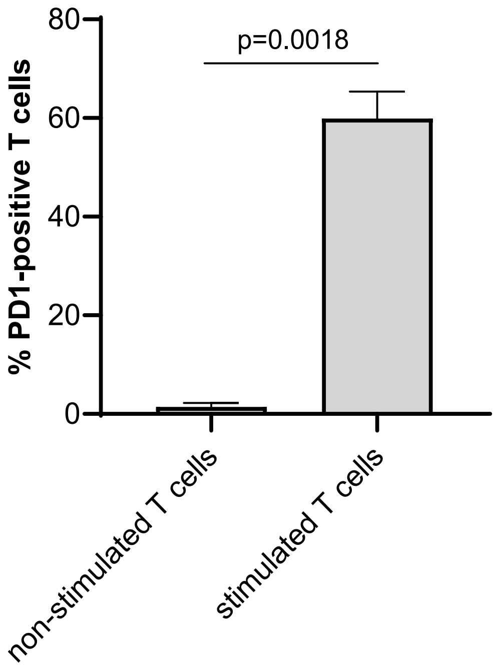

Figure S11. Flow cytometry analysis of PD1 expression in non-stimulated and stimulated T cells. The percentages of PD1-expressing T cells were measured by flow cytometry and are presented as means±SEM (n=4). Statistical analysis was performed by paired t-test.

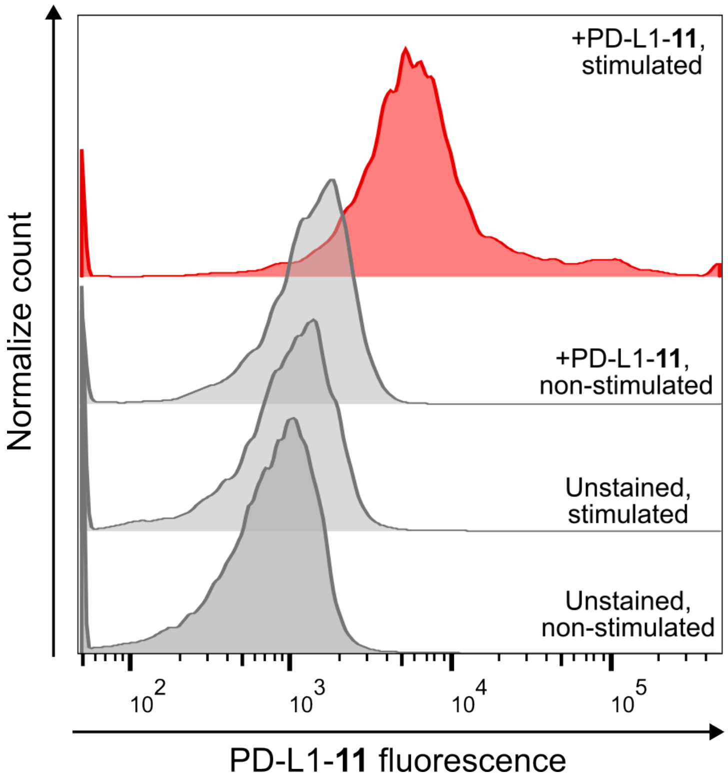

Figure S12. **Binding analysis of PD-L1-11 to non-stimulated and stimulated T cells.** Representative fluorescence histograms of non-stimulated (PD-1 negative) T cells and stimulated (PD-1 positive) T cells after incubation with **PD-L1-11** (5 µM) and flow cytometry analysis. Exc: 637 nm, em: 670±14 nm.

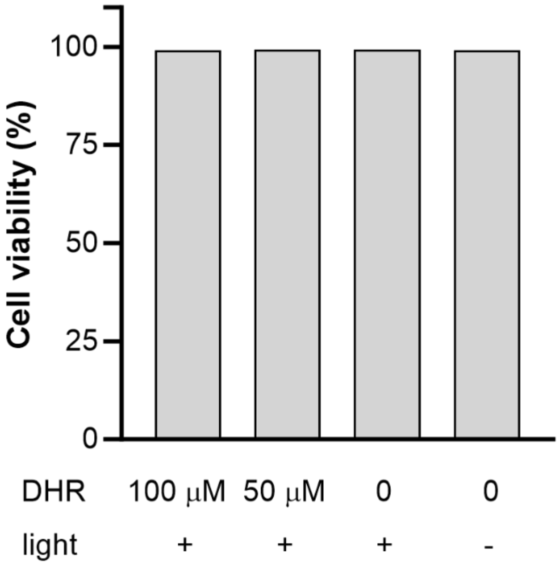

Figure S13. Viability of T cells under light irradiation. Cells were irradiated or not at 640 nm (10 mW cm^{-2}) for 60 min in the presence/absence of DHR. T cells were treated with Zombie Violet Live/Dead stain for 20 min at 4 °C followed by wash with PBS, and resuspended in FACS buffer (PBS+0.1% BSA) The percentages of live cells were determined by flow cytometry.

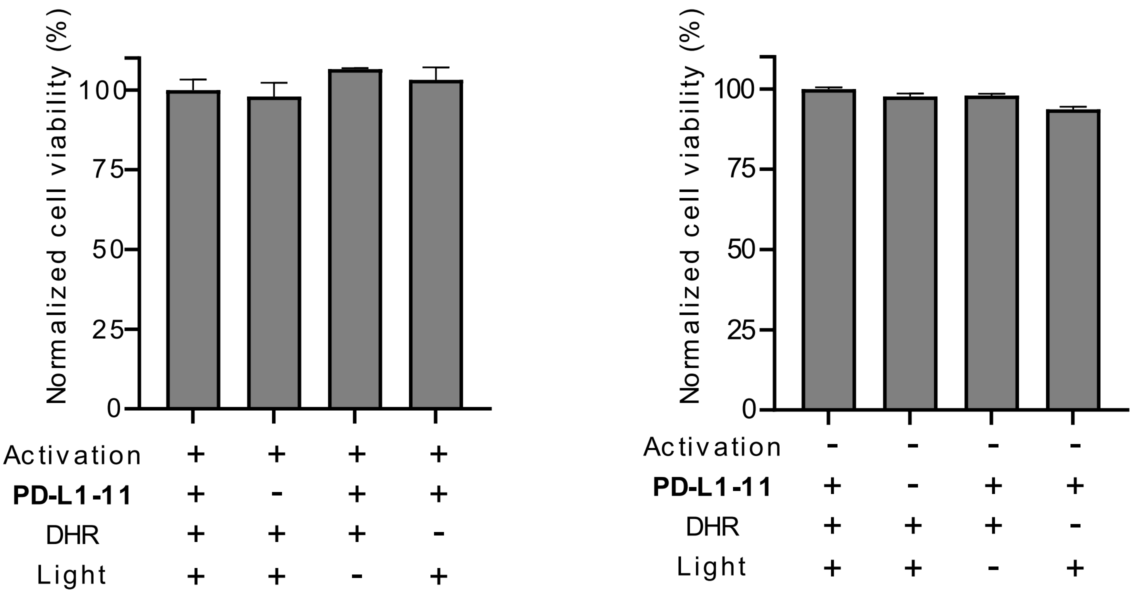

Figure S14. Viability of stimulated and non-stimulated T cells. Stimulated T cells (left) and non-stimulated T cells (right) were irradiated or not at 640 nm (10 mW cm^{-2}) for 60 min in the presence/absence of **PDL1-11** or DHR. Cell viability was determined by flow cytometry after incubation with the Zombie Violet Live/Dead stain for 20 min at 4 °C. Values are presented as means±SEM (n=3).

Table S1. Fluorescence and singlet oxygen quantum yields of compounds 5-9. 3,3'-Diethylthiadicarbocyanine iodide and methylene blue were used as references for the fluorescence quantum yields and singlet oxygen quantum yields, respectively. All measurements were performed in EtOH (n=3).

Compound	Φ_f (%)	Φ_Δ (%)
**5**	55±4	3.2±0.2
**6**	52±2	3.4±0.1
**7**	53±1	3.5±0.1
**8**	53±3	3.7±0.3
**9**	53±2	4.0±0.1

Table S2. HPLC purities of the compounds in the synthesis of pdCpA derivatives from compounds 5-7. High purities (>90%) were achieved in the synthesis of labelled pdCpA molecules using compounds **5-7** as precursors. The purity percentages were determined by HPLC-MS analysis.

	basic hydrolysis	pdCpA coupling	Boc removal
pdCpA-**5**	91%	>95%	>95%
pdCpA-**6**	>95%	>95%	>95%
pdCpA-**7**	>95%	>95%	91%

Table S3. Sequences of constructs used in this study.

Name	Sequence
NEB Express® backbone	TAATGAGGATCCCGGGAATTCTCGAGTAAGGTTAACCTGCAGG<br>AGGCCTTTAATTAAGGTGGTGCGGCCGCGCTAGCGGTCCCGG<br>GGGATCGATCCGGCTGCTAACAAAGCCCGAAAGGAAGCTGAG<br>TTGGCTGCTGCCACCGCTGAGCAATAACTAGCATAACCCCTTG<br>GGGCCTCTAAACGGGTCTTGAGGGGTTTTTTGCTGAAAGGAG<br>GAACTATATCCGGAAGCTTGGCACTGGCCGACCGGGGTCGAG<br>CACTGACTCGCTGCGCTCGGTCGTTCGGCTGCGGCGAGCGG<br>TATCAGCTCACTCAAAGGCGGTAATACGGTTATCCACAGAATCA<br>GGGGATAACGCAGGAAAGAACATGTGAGCAAAAGGCCAGCAA<br>AAGGCCAGGAACCGTAAAAAGGCCGCGTTGCTGGCGTTTTTC<br>CATAGGCTCCGCCCCCCTGACGAGCATCACAAAAATCGACGC<br>TCAAGTCAGAGGTGGCGAAACCCGACAGGACTATAAAGATACC<br>AGGCGTTTCCCCCTGGAAGCTCCCTCGTGCGCTCTCCTGTTC<br>CGACCCTGCCGCTTACCGGATACCTGTCCGCCTTTCTCCCTTC<br>GGGAAGCGTGGCGCTTTCTCATAGCTCACGCTGTAGGTATCTC<br>AGTTCGGTGTAGGTCGTTCGCTCCAAGCTGGGCTGTGTGCAC<br>GAACCCCCCGTTCAGCCCGACCGCTGCGCCTTATCCGGTAAC<br>TATCGTCTTGAGTCCAACCCGCTAAGACACGACTTATCGCCAC<br>TGGCAGCAGCCACTGGTAACAGGATTAGCAGAGCGAGGTATG<br>TAGGCGGTGCTACAGAGTTCTTGAAGTGGTGGCCTAACTACG<br>GCTACACTAGAAGAACAGTATTTGGTATCTGCGCTCTGCTGAA<br>GCCAGTTACCTTCGGAAAAAGAGTTGGTAGCTCTTGATCCGGC<br>AAACAAACCACCGCTGGTAGCGGTGGTTTTTTTGTTTGCAAGC<br>AGCAGATTACGCGCAGAAAAAAAGGATCTCAAGAAGATCCTTT<br>GATCTTTTCTACGGGGTCTGACGCTCAGTGGAACGAAAACTCA<br>CAGATCCGGGATTTTGGTCATGAGATTATCAAAAAGGATCTTCA<br>CCTAGATCCTTTTAAATTAAAAATGAAGTTTTAAATCAATCTAAA<br>GTATATATGAGTAAACTTGGTCTGACAGTTACCAATGCTTAATCA<br>GTGAGGCACCTATCTCAGCGATCTGTCTATTTCGTTCATCCATA<br>GTTGCCTGACTCCCCGTCGTGTAGATAACTACGATACGGGAGG<br>GCTTACCATCTGGCCCCAGTGCTGCAATGATACCGCGAGACC<br>CACGCTCACCGGCTCCAGATTTATCAGCAATAAACCAGCCAGC<br>CGGAAGGGCCGAGCGCAGAAGTGGTCCTGCAACTTTATCCGC<br>CTCCATCCAGTCTATTAATTGTTGCCGGGAAGCTAGAGTAAGTA<br>GTTCGCCAGTTAATAGTTTGCGCAACGTTGTTGCCATTGCTACA

	GGCATCGTGGTGTCACGCTCGTCGTTTGGTATGGCTTCATTCA GCTCCGGTTCCCAACGATCAAGGCGAGTTACATGATCCCCCAT GTTGTGCAAAAAAGCGGTTAGCTCCTTCGGTCCTCCGATCGTT GTCAGAAGTAAGTTGGCCGCAGTGTTATCACTCATGGTTATGG CAGCACTGCATAATTCTCTTACTGTCATGCCATCCGTAAGATGC TTTTCTGTGACTGGTGAGTACTCAACCAAGTCATTCTGAGAATA GTGTATGCGGCGACCGAGTTGCTCTTGCCCGGCGTCAATACG GGATAATACCGCGCCACATAGCAGAACTTTAAAAGTGCTCATCA TTGGAAAACGTTCTTCGGGGCGAAAACTCTCAAGGATCTTACC GCTGTTGAGATCCAGTTCGATGTAACCCACTCGTGCACCCAAC TGATCTTCAGCATCTTTTACTTTCACCAGCGTTTCTGGGTGAGC AAAAACAGGAAGGCAAAATGCCGCAAAAAAGGGAATAAGGGC GACACGGAAATGTTGAATACTCATACTCTTCCTTTTTCAATATTA TTGAAGCATTTATCAGGGTTATTGTCTCATGAGCGGATACATATT TGAATGTATTTAGAAAAATAAACAAATAGGGGTTCCGCGCACAT TTCCCCGAAAAGTGCTAGTGGTGCTAGCCCCGCGAAATTAATA CGACTCACTATAGGGTCTAGAAAT
PD-L1 gene insert	CGAAATTAATACGACTCACTATAGGGTCTAGAAATAATTTTGTTT AACTTTAAGAAGGAGATATACATGTTTACTGTAACAGTCCCTAAA GACTTATATGTTGTCGAGTACGGCTCTAACATGACAATCGAGTG TAAGTTTCCGGTAGAGAAGCAGCTTGACTTAGCTGCTTTGATT GTGTACTGGGAGATGGAAGATAAGAATATAATTCAGTTCGTCCA TGGAGAAGAGGACTTGAAAGTACAGCACAGTAGTTACCGGCA GCGGGCGAGATTGTTAAAGGATCAGTTAAGTCTTGGTAACGCT GCACTTCAAATAACTGATGTTAAATTACAGGACGCTGGCGTTTA CCGCTGTATGATCTCATACGGTGGCGCCGACTACAAACGTATA ACCGTAAAGGTGAATGCACCCTACAACAAGATTAATCAACGCAT CTTGGTGGTGGACCCAGTTACCTCGGAGCATGAATTGACATGT CAGGCAGAGGGGTATCCTAAGGCAGAGGTGATATGGACTTCG TCCGATCATCAAGTATTGTCAGGTAAAACAACAACGACGAACA GCAAAAGAGAAGAAAAGCTGTTCAATGTGACTAGCACGCTTCG GATTAACACTACTACAAATGAAATTTTTTACTGTACGTTTAGACG GTTAGATCCTGAGGAGAACCATACTGCCGAGCTTGTTATACCA GAATTGCCGCTTGCCCATCCTCCCAATGAGCGGACGCACCAT CACCACCATCATGGAAGCGGCGGTTCTGGTGGTGGTTCTGGT TAATGAGGATCCCGGGAATTCTCGAGTAAG
IL-33 gene	CGAAATTAATACGACTCACTATAGGGTCTAGAAATAATTTTGTTT AACTTTAAGAAGGAGATATACATGAAATCCCTGATTACGCCTATC

insert	GCTGCTGGGTTGCTTCTGGCGTTCTCTCAATATTCGCTTGCAT<br>CGTCAATCACAGGAATCTCCCCGATTACGGAATATTTAGCGTCA<br>TTGTCCACCTATAACGATCAATCCATTACATTTGCGCTGGAGGA<br>TGAGTCGTATGAGATTTATGTTGAGGACTTAAAGAAGGACGAG<br>AAGAAAGACAAAGTGCTGTTGTCCTACTACGAGTCTCAACACC<br>CGTCGAATGAGTCGGGGGACGGGGTAGACGGCAAGATGTTAA<br>TGGTGACTCTGTCTCCGACTAAAGACTTTTGGTTGCACGCTAA<br>CAATAAAGAACACTCCGTTGAGTTGCACAAGAGCGAAAAGCCT<br>CTTCCCGATCAAGCGTTCTTCGTGTTACACAACATGCACTCTAA<br>TAGCGTCAGCTTCGAGAGCAAGACAGATCCGGGCGTCTTTATC<br>GGAGTCAAAGATAACCACCTGGCATTGATCAAAGTTGACTCTT<br>CGGAAAACCTTTCTACAGAGAATATCTTATTTAAACTGAGCGAA<br>ACACACCATCACCACCATCATGGAAGCGGCGGTTCTGGTGGT<br>GGTTCTGGTTAATGAGGATCCCGGGAATTCTCGAGTAAG

4. NMR Spectra

Compound **1** ($CDCl_3$)

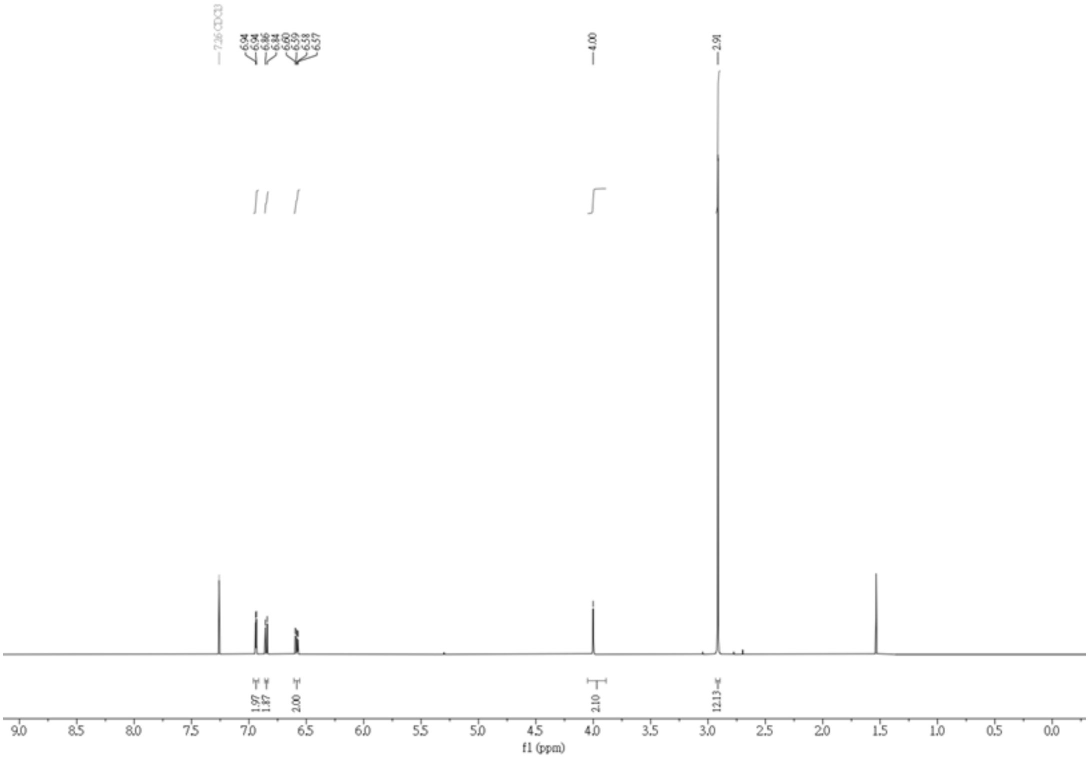

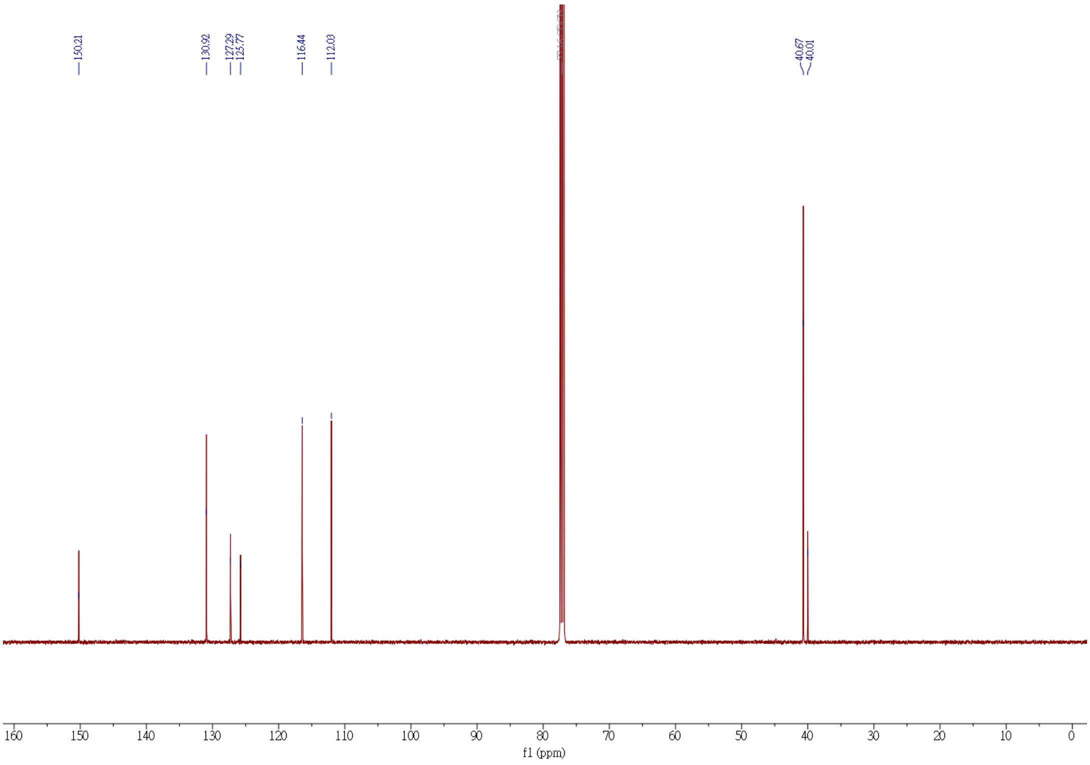

Compound **2** ($CDCl_3$)

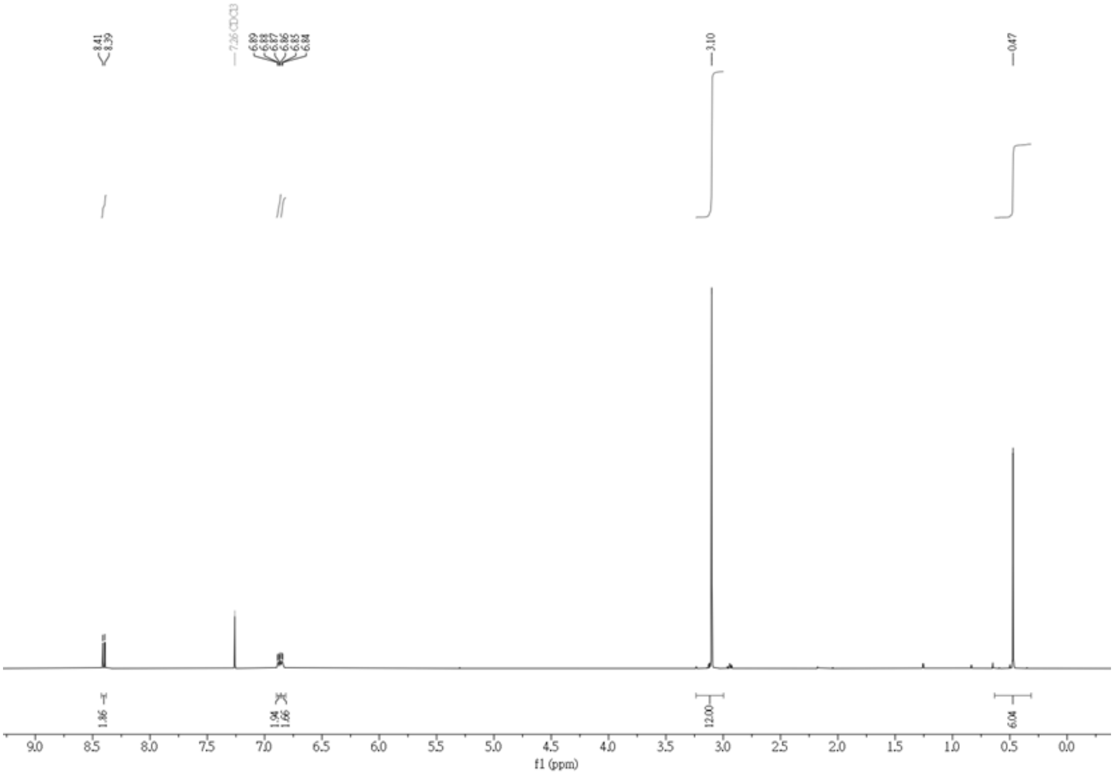

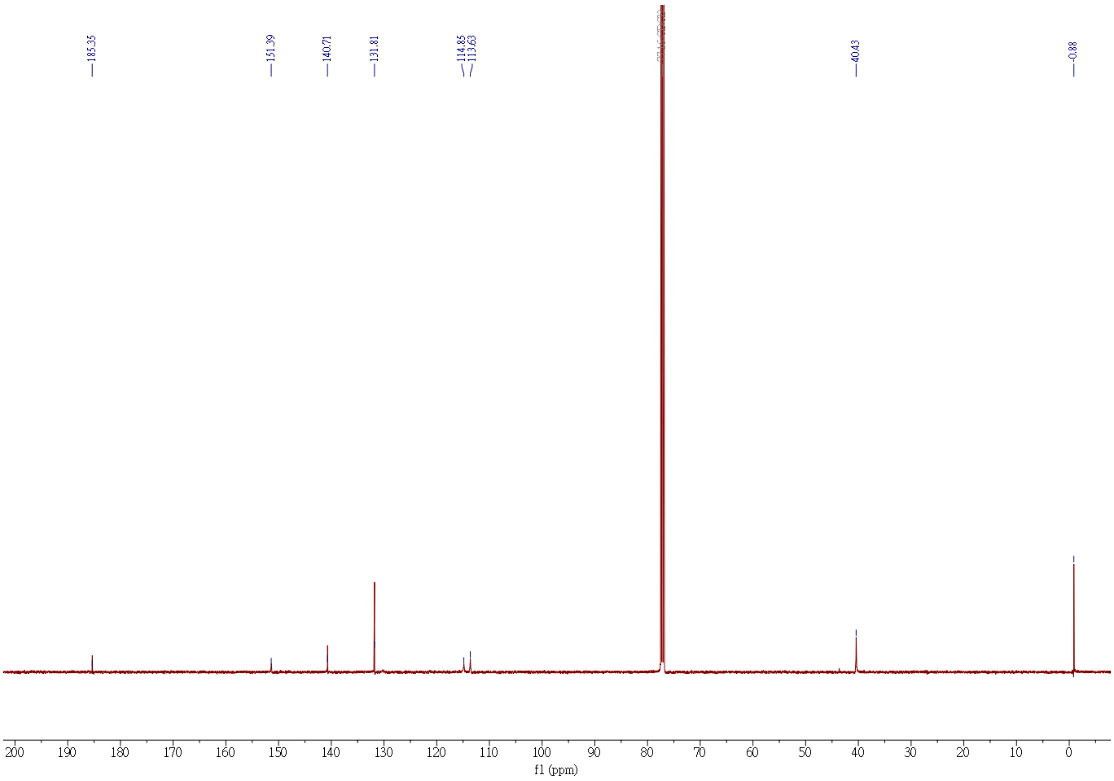

Compound **3** ($CDCl_3$)

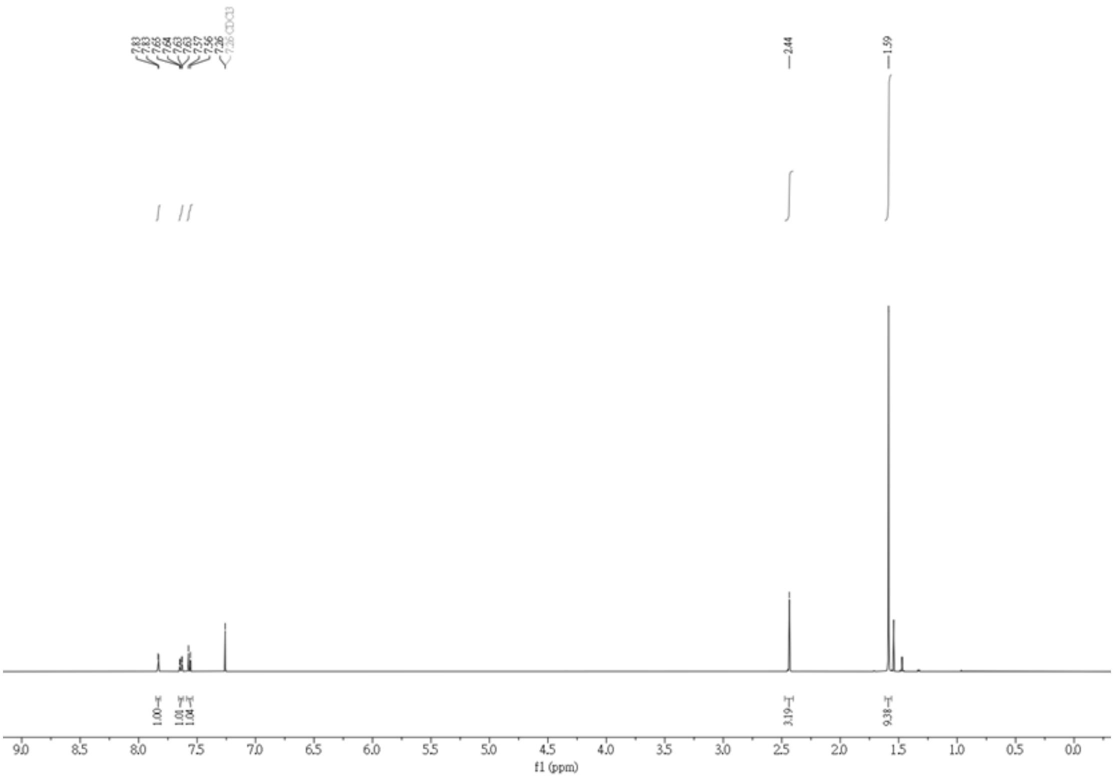

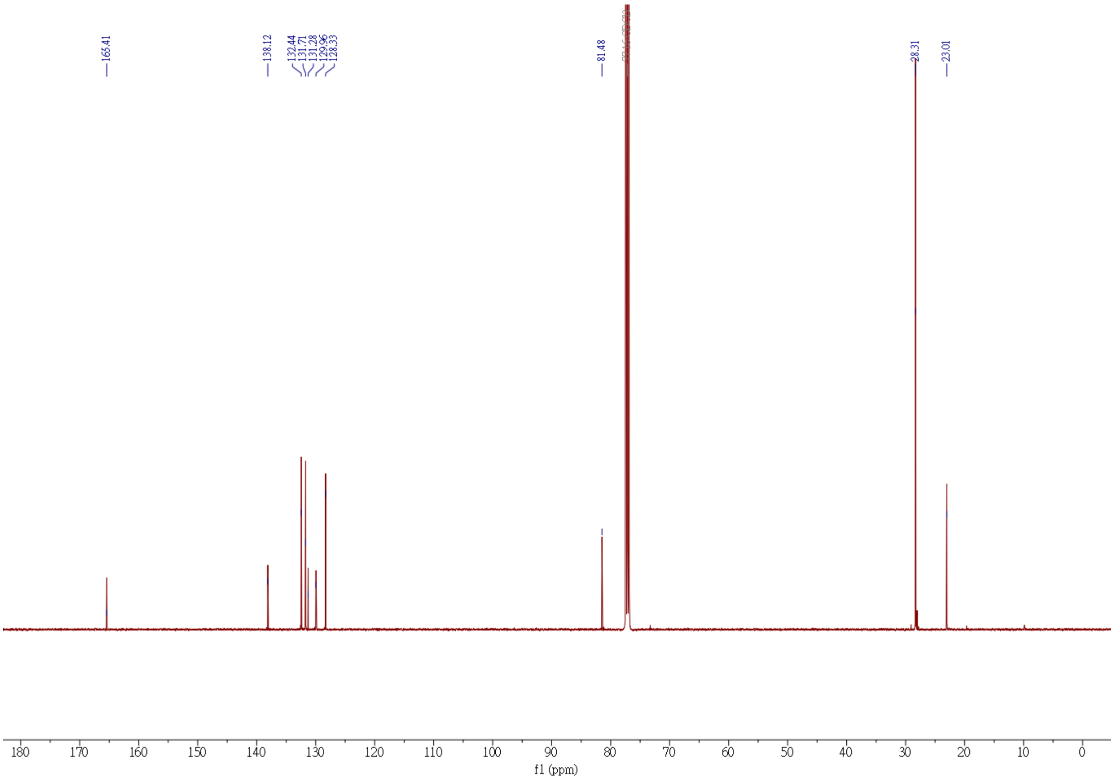

Compound **4** ($CDCl_3$)

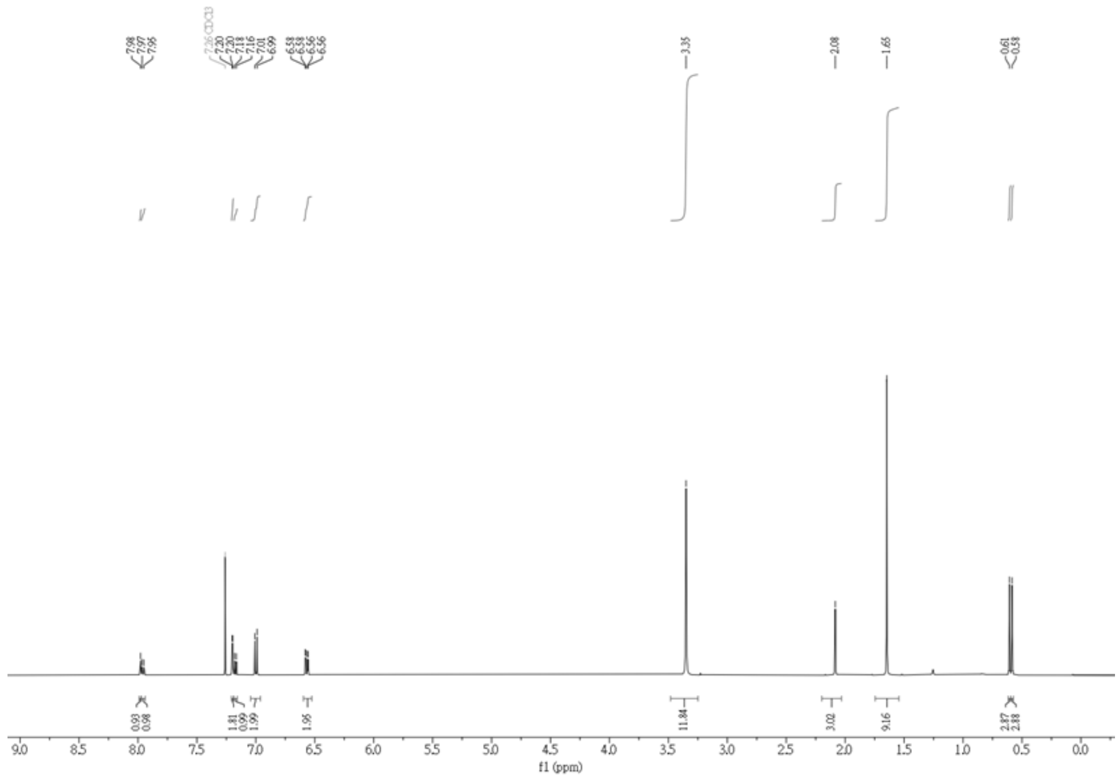

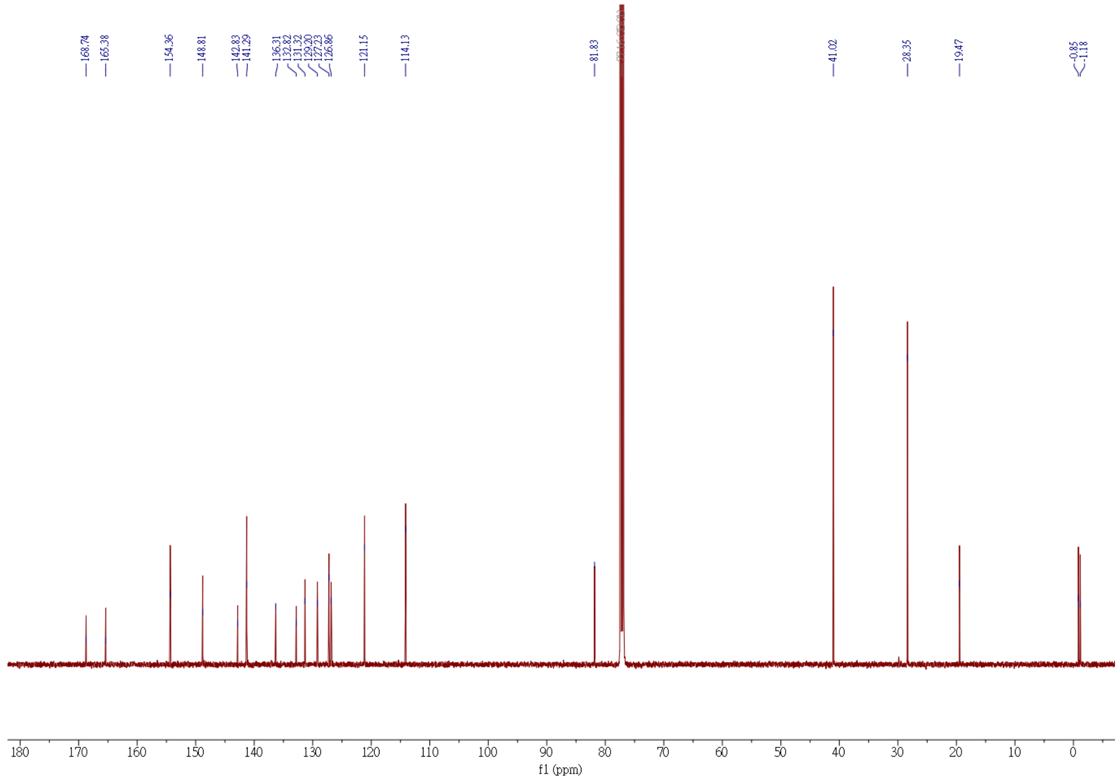

Compound **5** ($CDCl_3$)

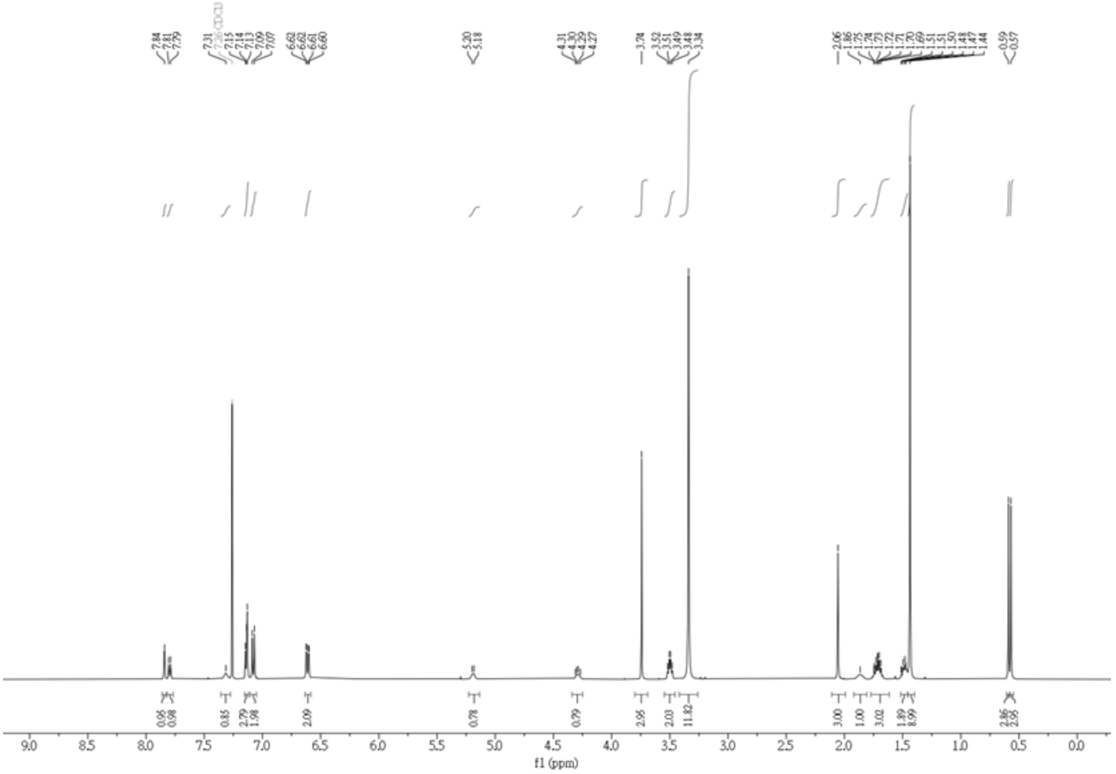

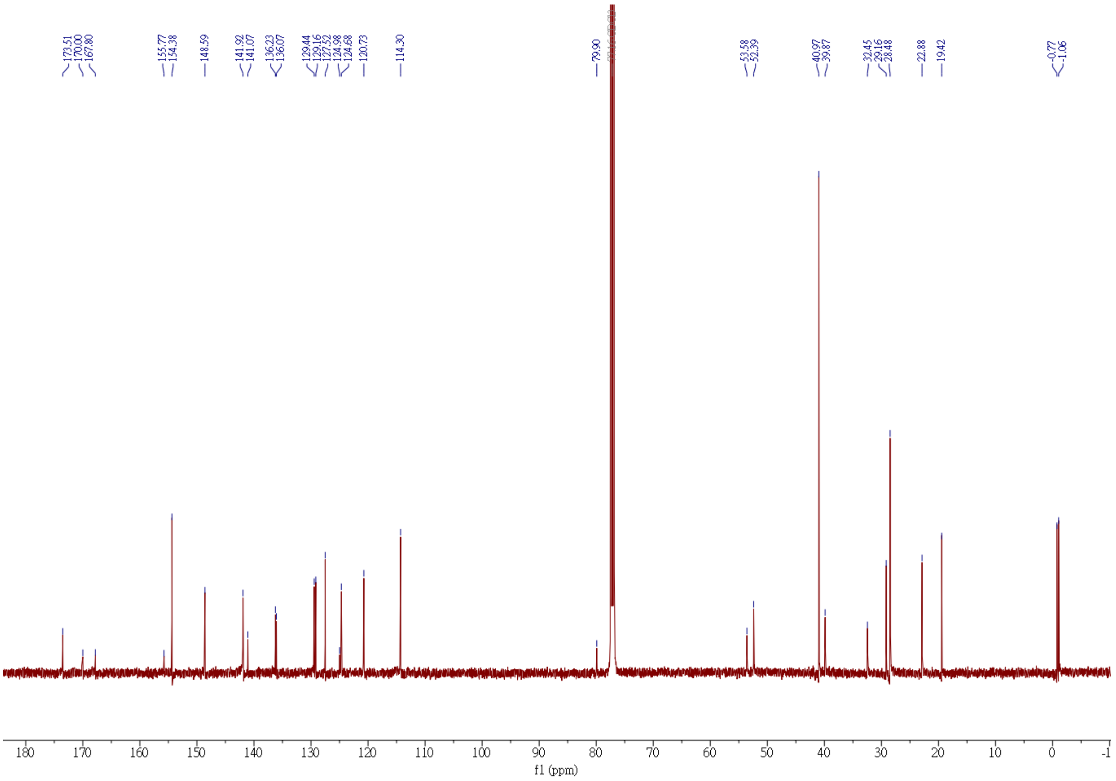

Compound **6** ($CDCl_3$)

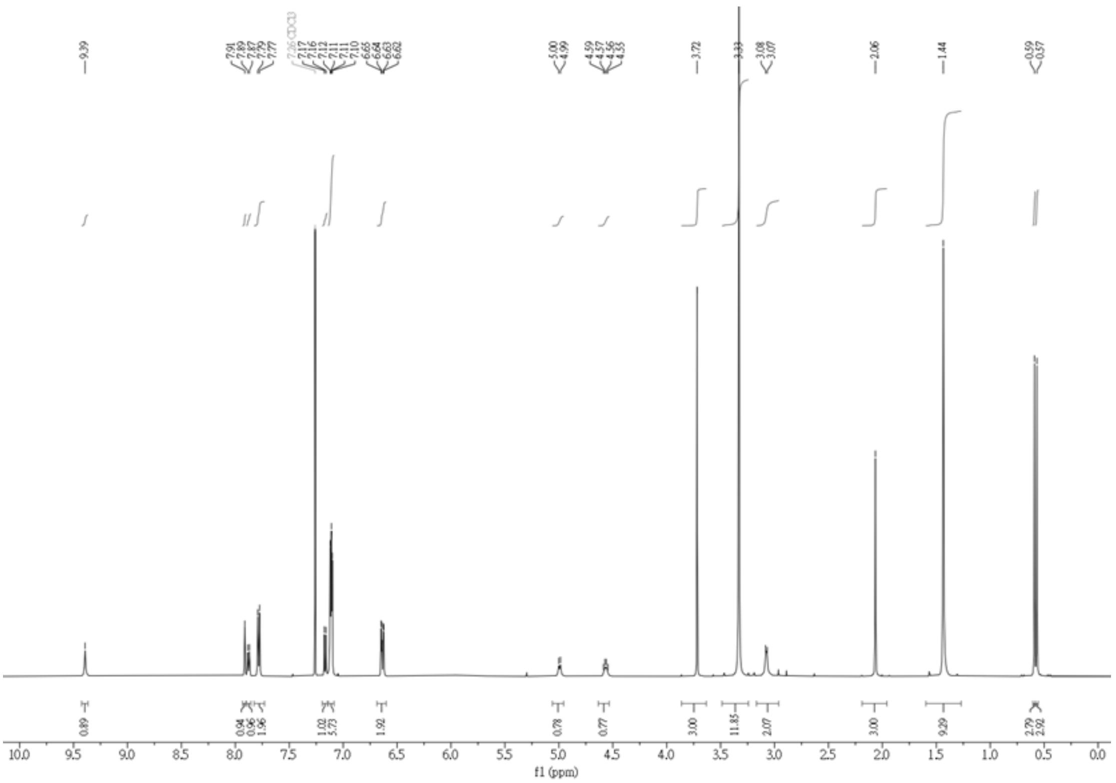

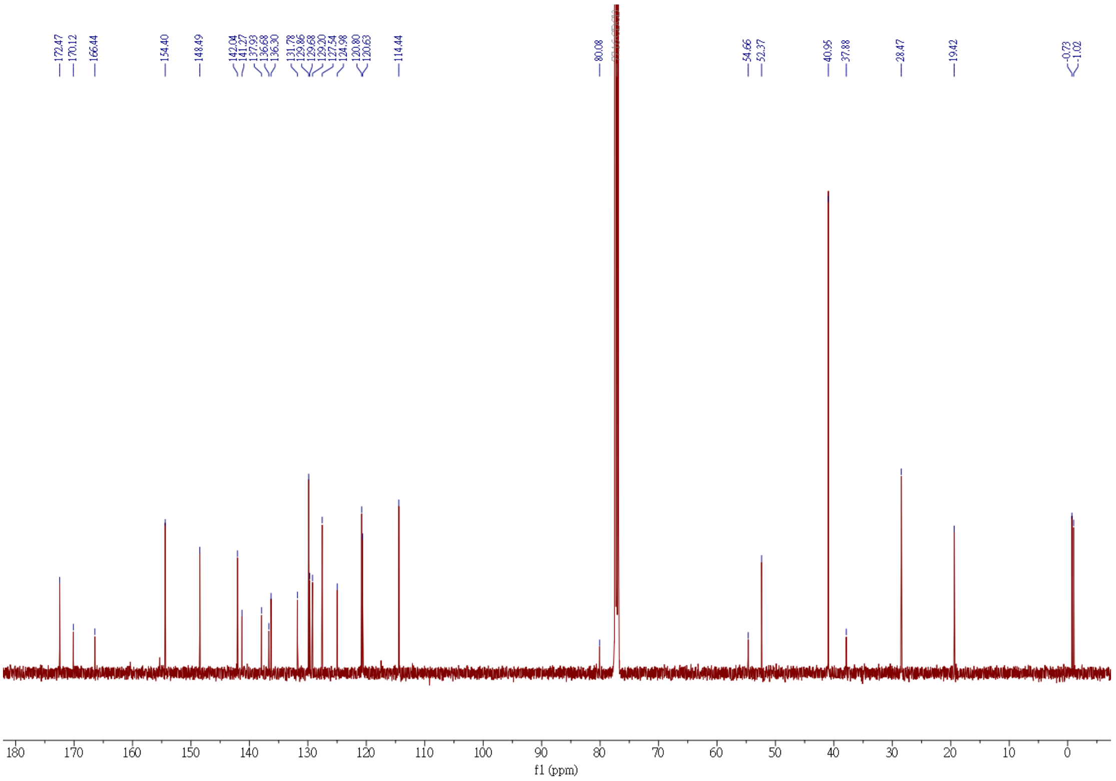

Compound **7** ($CDCl_3$)

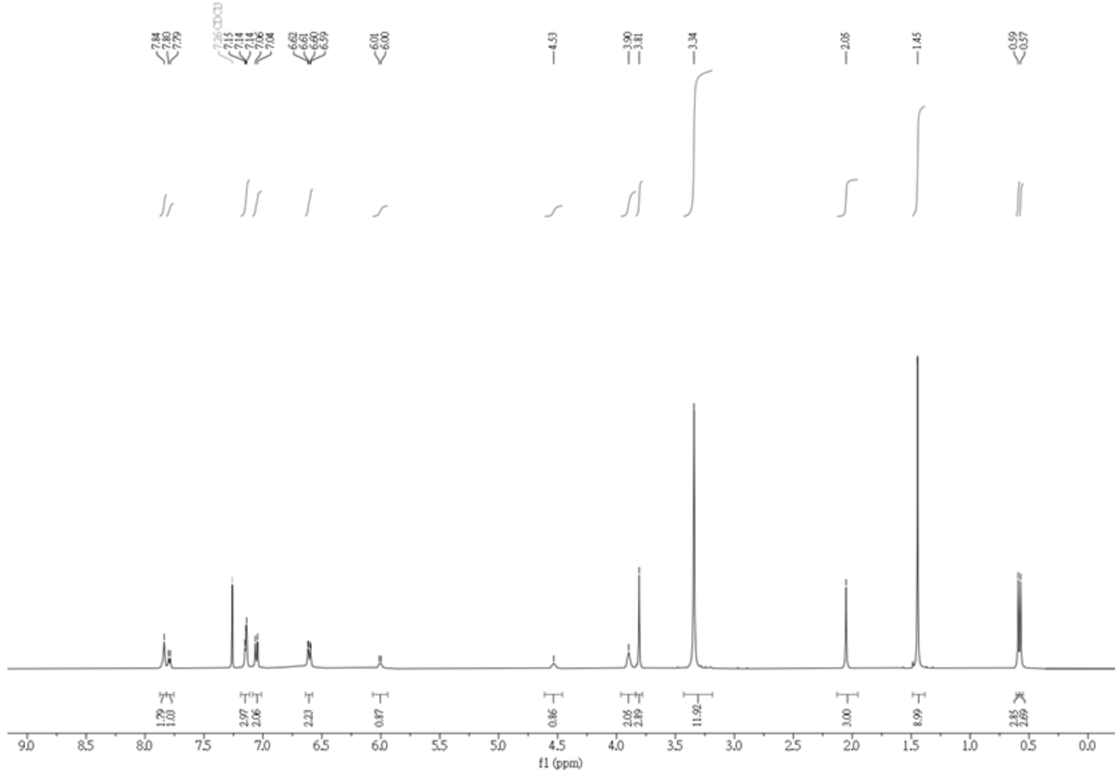

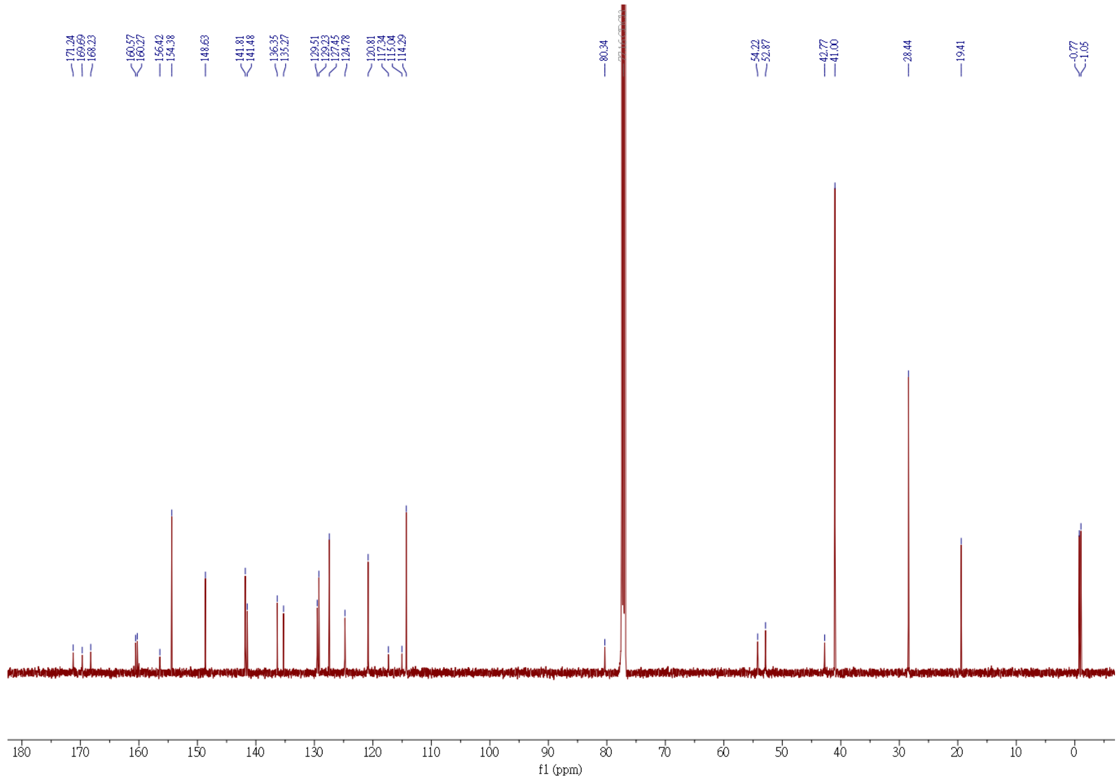

Compound **8** ($CDCl_3$)

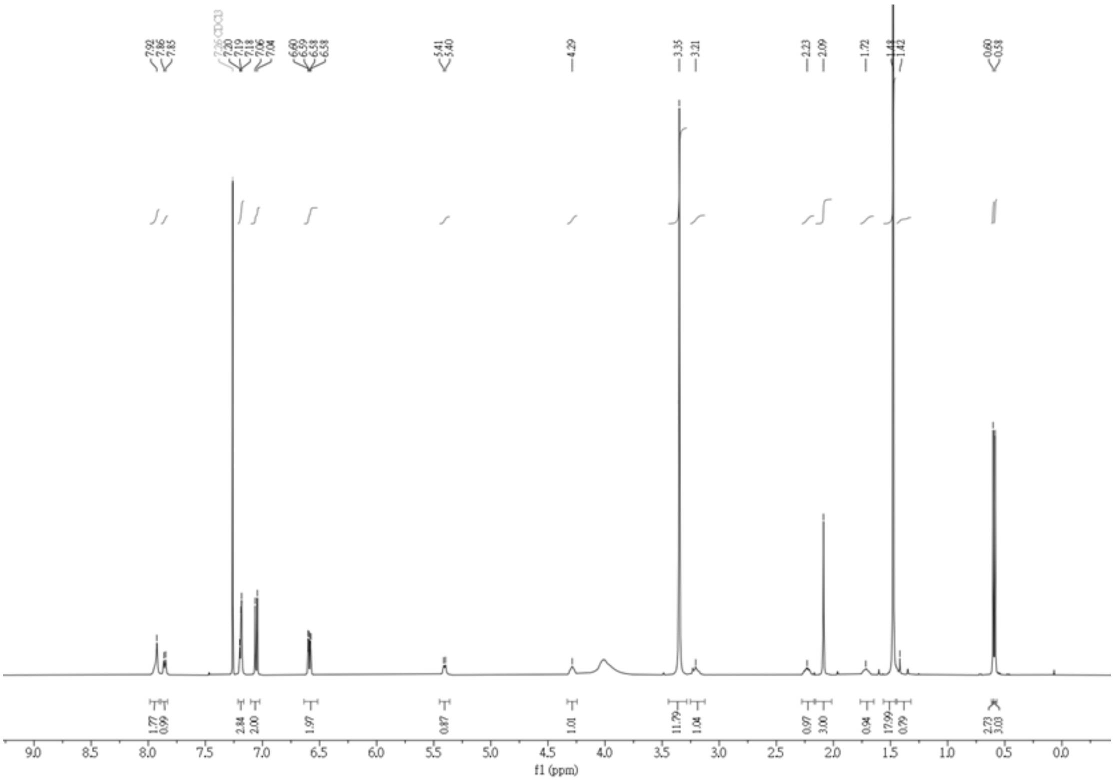

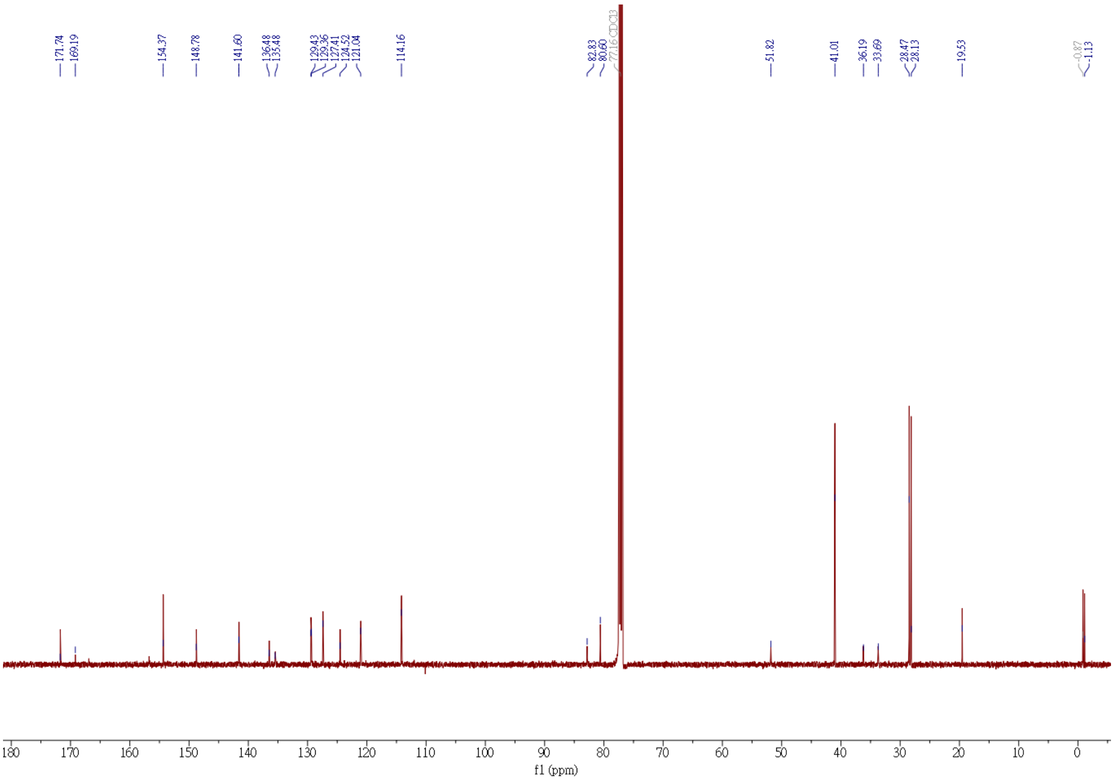

Compound **9** ($CDCl_3$)

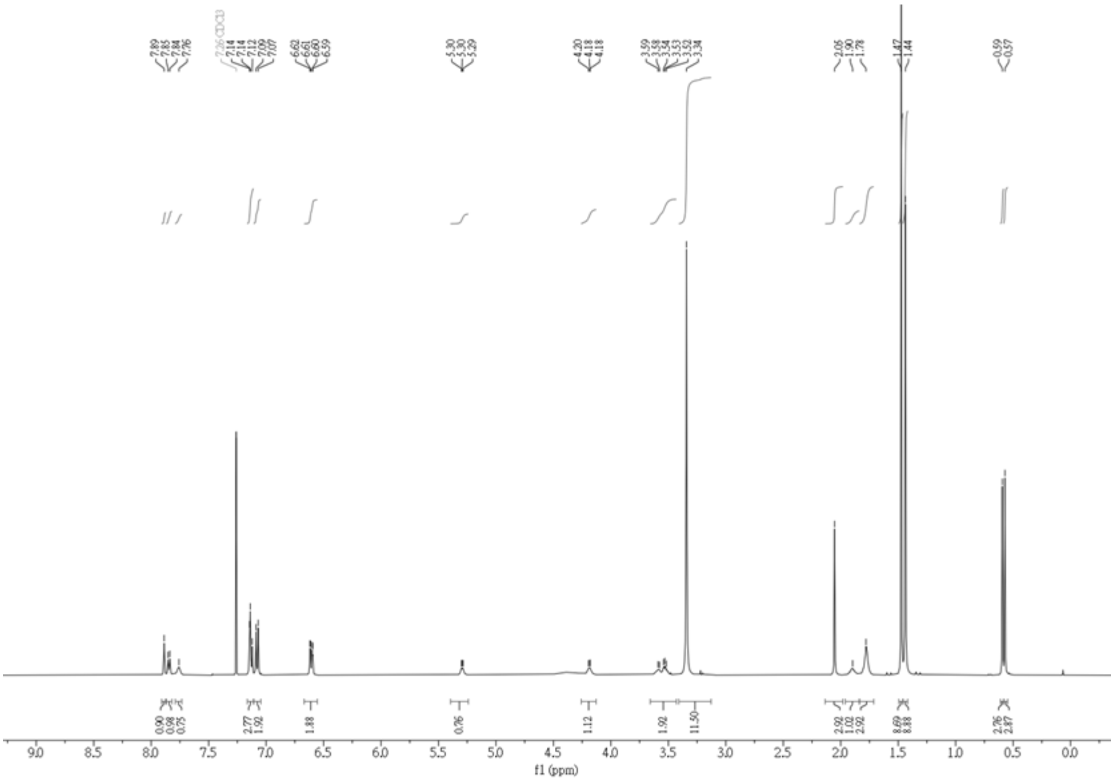

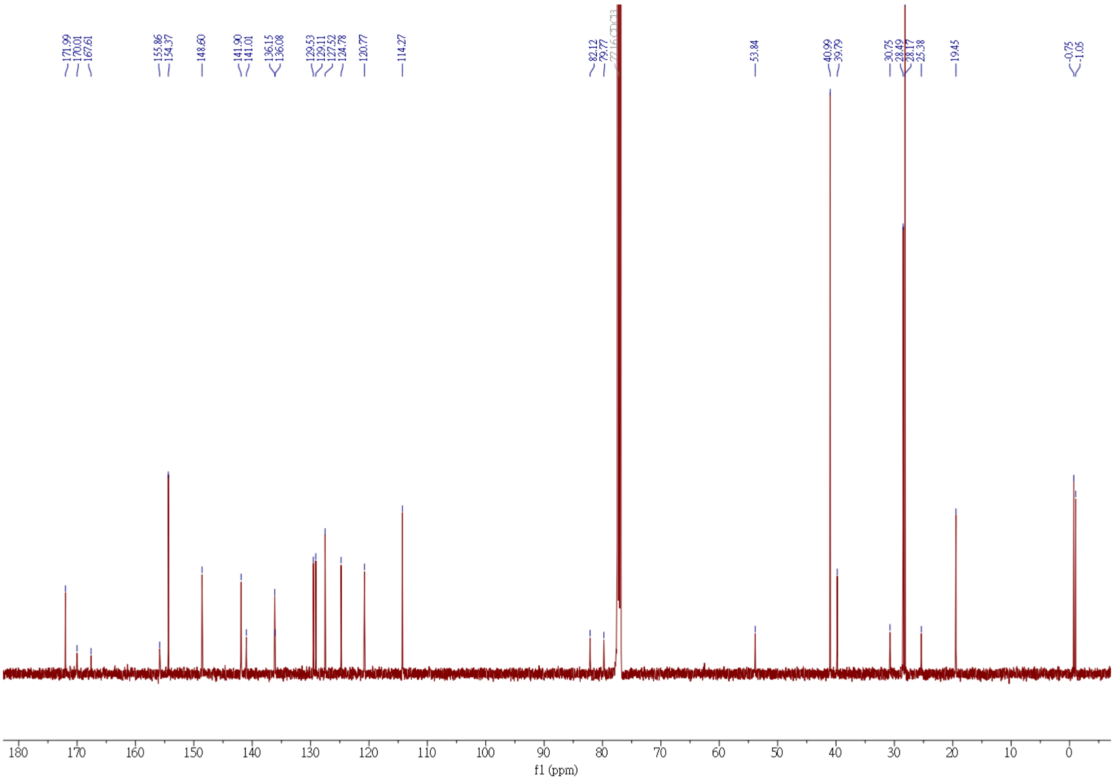

Compound **10** ($CDCl_3$)

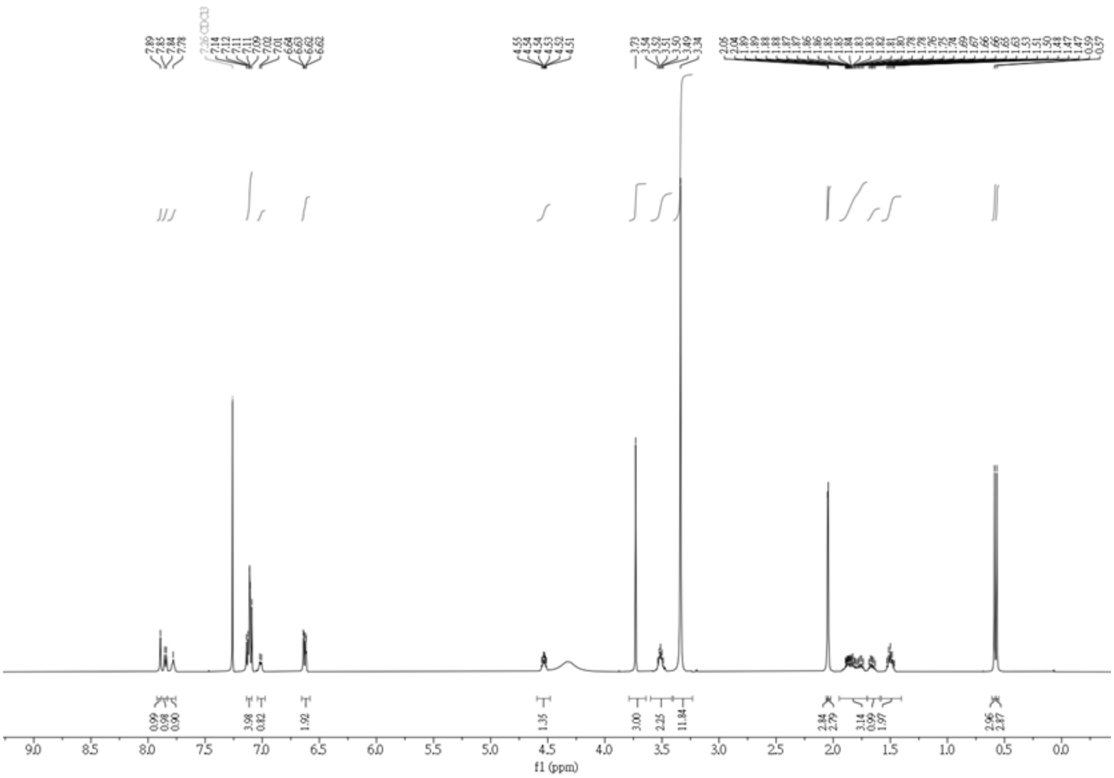

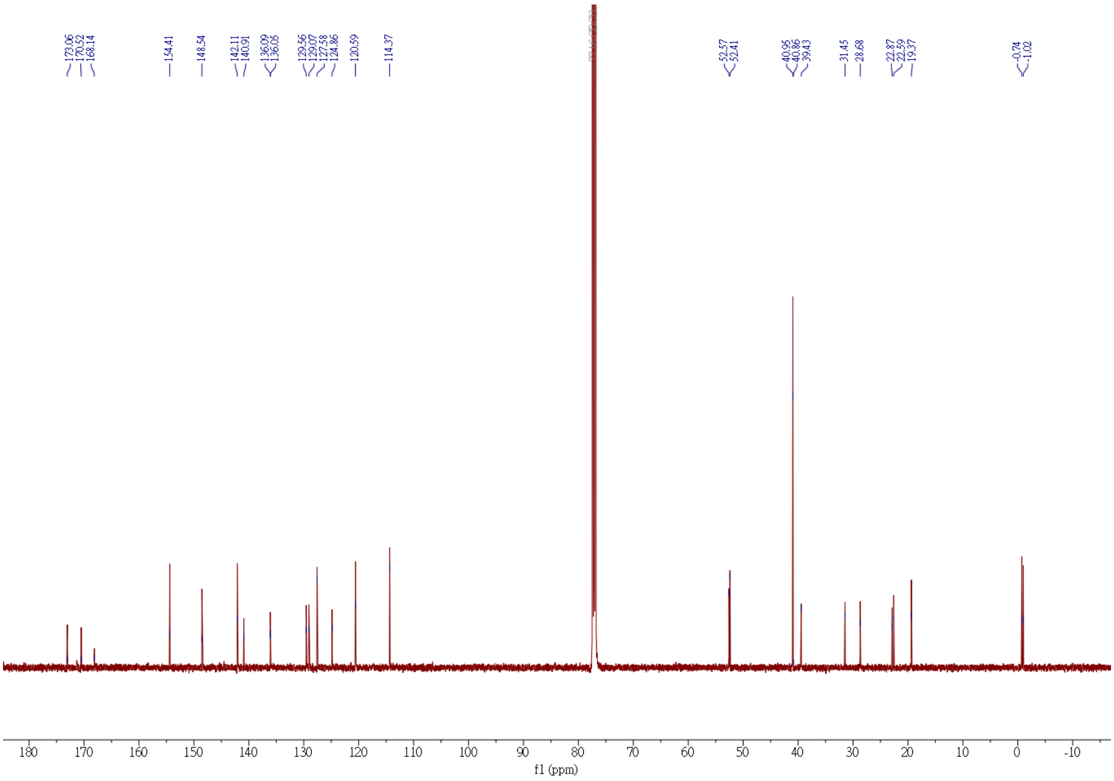

Compound **11** ($CDCl_3$)

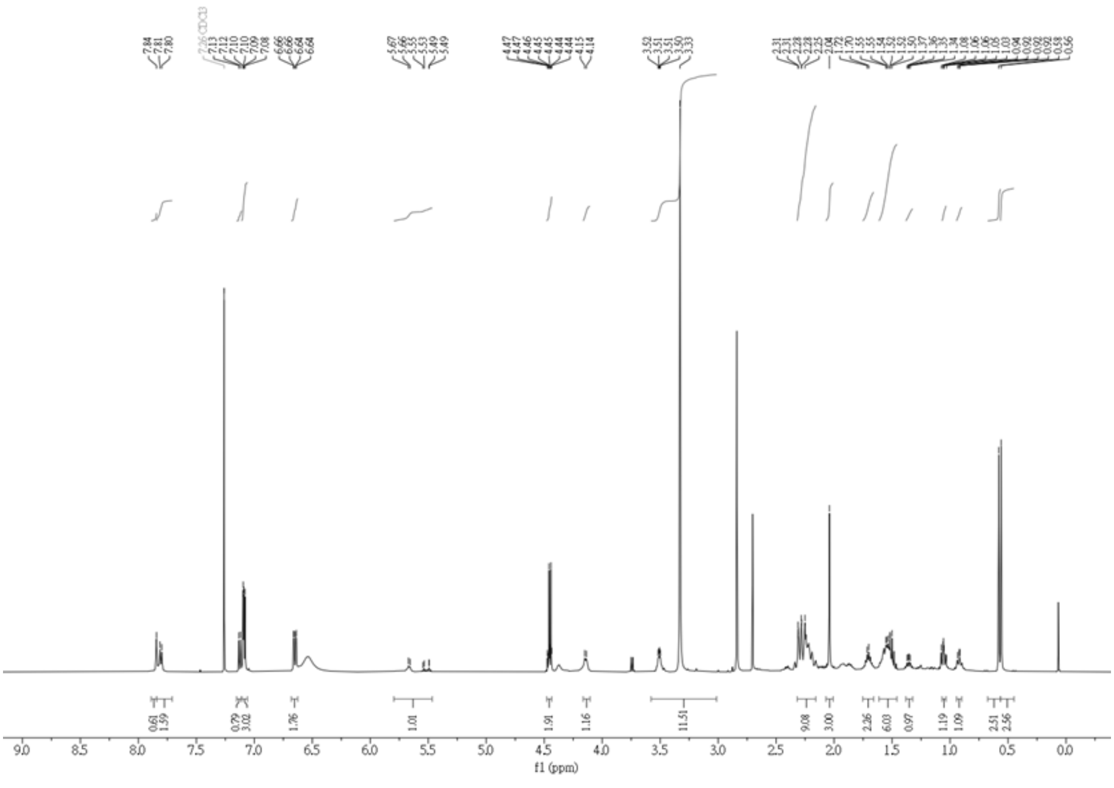

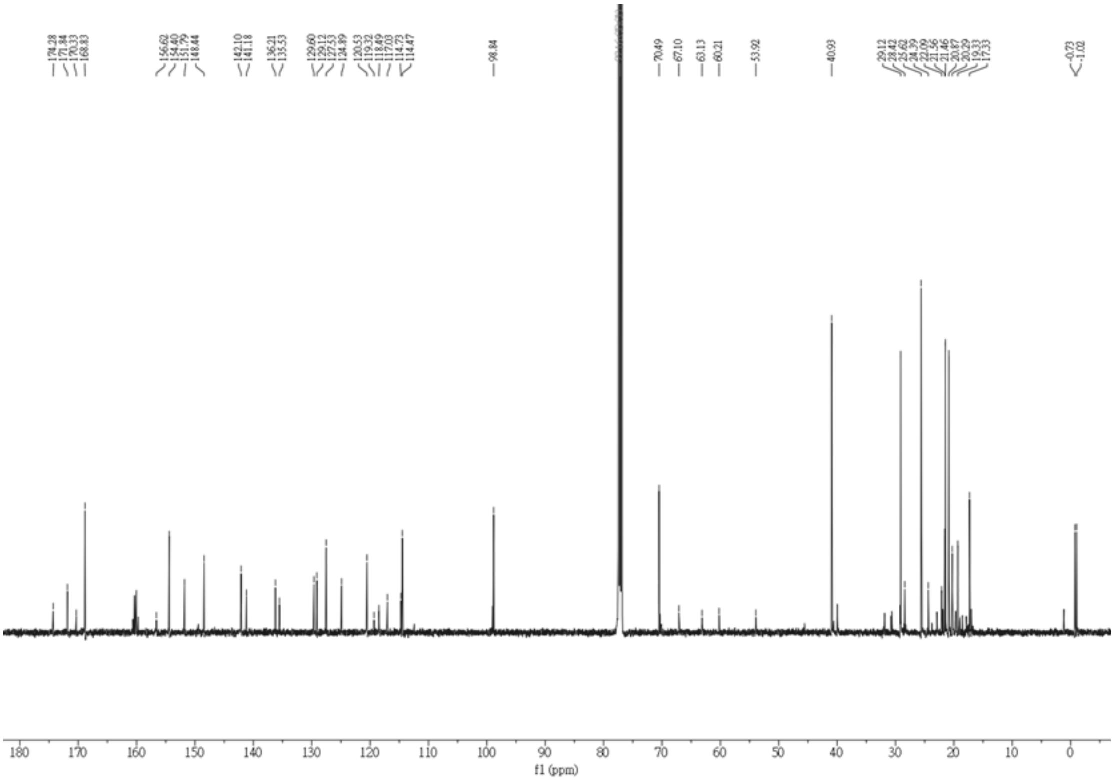

5. Supplementary References

[1] R. Sato, et al. *J. Am. Chem. Soc.* 2017, **139**, 17397.

[2] M. Ye, et al. *Adv. Mat.* 2017, **29**, 1702342.

[3] S. Benson, et al. *Nat. Commun.* 2021, **12**, 2369.
